# Proteome Signature of Alzheimer-Like Phenotypes in Frontal Cortices From Young and Old Individuals With Down Syndrome

**DOI:** 10.1007/s12035-025-05432-0

**Published:** 2025-11-21

**Authors:** Fabio Di Domenico, Viviana Greco, Antonella Tramutola, Monika Rataj-Baniowska, Eugenio Barone, Chiara Lanzillotta, Luisa Pieroni, D. Allan Butterfield, Yann Herault, Sara Pagnotta, Tommaso Cassano, Elizabeth Head, Andrea Urbani, Marzia Perluigi

**Affiliations:** 1 Department of Biochemical Sciences A. Rossi Fanelli, Laboratoryaffiliated to , Istituto Pasteur Italia-Fondazione Cenci Bolognetti, Sapienza University of Rome, P.Le Aldo Moro 5, 00185 Rome, Italy; 2 Department of Basic Biotechnological Sciences, Intensivological and Perioperative Clinics, Università Cattolica del Sacro Cuore, 00168 Rome, Italy; 3 Department of Laboratory Diagnostic and Infectious Diseases, Unity of Chemistry, Biochemistry and Clinical Molecular Biology, Fondazione Policlinico Universitario Agostino Gemelli-IRCCS, 00168 Rome, Italy; 4 Institut de Génetique Biologie Moléculaire Et Cellulaire, Université de Strasbourg, CNRS, IGBMC, 7104- -S 1258, F-67400 Illkirch, InsermUMR France; 5 UniCamillus-Saint Camillus International University of Health and Medical Sciences, Via Di Sant’Alessandro, 8, 00131 Rome, Italy; 6 IRCCS San Camillo Hospital, Via Alberoni, 70, 30126 Lido, Venice, Italy; 7 Department of Chemistry, University of Kentucky, Lexington, KY 40506 USA; 8 Department of Medical and Surgical Sciences, University of Foggia, Via Antonio Gramsci, 89, 71122 Foggia, Italy; 9 Department of Pathology & Laboratory Medicine, University of California, Irvine, CA 94143 USA; 10 Sanders-Brown Centre On Aging, University of Kentucky, Lexington, KY 40536 USA

**Keywords:** Mass spectrometry, Alzheimer’s disease, Proteome, Trisomy 21, Mouse model, Ts66Yah

## Abstract

Down syndrome (DS) stands out as the most prevalent genetic contributor to intellectual disability, marked by the presence of an extra copy of chromosome 21 (HSA21). Notably, individuals with DS exhibit significant neuropathological changes for a diagnosis of Alzheimer’s disease (AD), typically by the age of 50 years. To search for and identify biomarkers crucial for detecting and understanding the mechanisms involved in DS neuropathology, we conducted a protein expression analysis of post-mortem brain samples. We evaluated the frontal cortex of post-mortem brain samples from patients with DS both before and after the onset of AD pathology (DSAD), in comparison with age-matched healthy patients (CTRY and CTRO). Employing a comprehensive label-free shotgun proteomics approach, we sought to gain a deeper understanding of the intricate protein profiles associated with DS and its progression into DSAD. Collected results have been analyzed using specific databases and bioinformatics analysis software to understand relevant pathways, networks, and functions related to the experimental data. Our data support a genotype effect in DS at young and old ages that promotes specific proteome signatures associated with AD development. Notably, the affected signalling pathways encompass energy-related processes, synaptic transmission, and stress response. With aging, the dynamic shift in protein expression contributes to accelerating the neurodegenerative process, culminating in the manifestation of the AD phenotype.

**Supplementary Information:**

The online version contains supplementary material available at 10.1007/s12035-025-05432-0.

## Introduction

Down syndrome (DS), or trisomy 21, is the most common genetic cause of intellectual disability with an incidence of approximately 1/1000 births. People with DS present with diverse phenotypes, although individual variability occurs [1]. Common features of DS involve facial abnormalities and skeletal disorders, congenital heart defects and hematological complications, gastrointestinal abnormalities, and neurological disorders. The latter represents a crucial feature of the DS phenotype that is associated with accelerated aging and the risk of developing early-onset Alzheimer’s disease (EOAD). Studies in post-mortem human brain from individuals with DS and mouse models of DS identify several dysfunctional processes in neuronal cells, including growth, differentiation, oxidative stress, myelination, and synaptic function [2]. All these disturbances translate into a clinical phenotype characterized by cognitive and language dysfunction coupled with sensory and neuromotor deficits and, later in life, AD neuropathology.

A defining consequence of the trisomy underlying DS is the altered expression of genes located on human chromosome 21 (HSA21), driven by genome dosage imbalance. Several studies have shown that the pathological features of DS arise from complex, large-scale transcriptomic alterations triggered by the genomic imbalance of HSA21, in conjunction with changes in disomic gene expression. Transcriptomic analyses have been employed to identify dosage-sensitive HSA21 genes [3], and transcriptome profiles are now available for various human DS tissues as well as for mouse models of the disorder [4–8]. Collectively, these studies underscore the critical impact of gene dosage effects on HSA21 genes while also revealing widespread secondary transcriptional changes across the entire genome. However, the landscape is further complicated by inconsistencies among studies regarding which trisomic genes exhibit increased expression and the extent of their upregulation. The expression of trisomic genes appears to be dynamic, and mechanisms such as dosage compensation may account for why not all HSA21 genes show elevated expression despite the presence of an extra chromosome copy. Moreover, whether dosage compensation is substantial may depend on the context (i.e., tissue or developmental stage) [9]. Interestingly, studies from Lockstone et al*.* characterized, by whole genome microarrays, the transcriptome of human adult brain tissue from DS vs. age-matched controls [10]. Functional profiling of genes dysregulated in both fetal and adult datasets identifies categories including development, lipid transport, and cellular proliferation. In the adult brain, these processes were concomitant with cytoskeletal regulation and vesicle trafficking categories and increased immune response and oxidative stress response, which are likely linked to the development of AD pathology in individuals with DS.

However, trisomy of protein-coding genes and gene dosage-associated increases in RNA expression do not directly correlate with corresponding elevation in protein expression [11]. Moreover, given that proteins are the primary effectors of cellular function, any contribution of a triplicated gene to the DS phenotype ultimately depends on alterations in its corresponding protein levels, highlighting the importance of proteomic measurements. While gene dosage is a key factor, additional regulatory mechanisms, such as epigenetic modifications, can influence both the transcription and translation of HSA21 genes, thereby modulating their expression and potentially mitigating overexpression [12]. In the present study, we examined the complex proteome signature in DS, before and after the development of AD neuropathology. We analyzed the frontal cortex of individuals with DS and those with DS affected by AD (DSAD), comparing them with their respective age-matched controls. By comparing data across all four groups, we identified protein categories that exhibited significant alterations in individuals with DS relative to age-matched controls. Interestingly, young individuals with DS exhibited a general trend of increased protein upregulation, affecting products of both trisomic and disomic genes. Conversely, an overall reduction in protein expression levels was observed in the aged groups, both in controls and DSAD individuals, with the latter exhibiting a more pronounced decline.

In conclusion, the current study contributes to shedding light on the intricate relationship among gene-protein-phenotype by showing how extensive protein remodelling, caused by aneuploidy, dynamically occurs through life in DS subjects, resulting in significant modulation and severity of phenotypes.

## Materials and Methods

### Human Brain Samples

Frontal cortex human brain samples were obtained from the University of California Alzheimer’s Disease Research Center (UCI-ADRC) and the Institute for Memory Impairments and Neurological Disorders, the Eunice Kennedy Shriver NICHD Brain and Tissue Bank for Developmental Disorders, and the University of Kentucky Alzheimer’s disease Center. Table 1 (and Sup. Table 1) shows the characteristics and demographic data of the included cases in the study. DS cases were divided into two groups, with (DSAD, *n* = 6) or without (DS, *n* = 6) sufficient neuropathology for diagnosis of AD. The autopsy cases included in this study were analyzed by Cenini and colleagues for amyloid beta (Aβ) deposition, demonstrating an increase of the soluble and insoluble forms, and of oligomers in the frontal cortex from young and old DS individuals as a function of age and of AD development [13]. Furthermore, post-mortem cortical samples were previously analyzed for Tau pathology, protein oxidation markers, autophagy pathway, and insulin signalling, demonstrating, as well, significant alterations in DS since young age [14–16].

**Table 1:** Patient’s demographic data

Subjects	PMI	Age	Sex	Ethnicity
CTRY	**9.96 ± 2.88**	**24.9 ± 9.95**	**4 M, 2 F**	**1 AA, 4 Ca, 1 Un**
DS	**12.5 ± 1.51**	**26.7 ± 16.8**	**4 M, 2 F**	**1 AA, 4 Ca, 1 In**
DSAD	**5.4 ± 2.8**	**59.3 ± 3.44**	**2 M, 4 F**	**6 Un**
CTRO	**8.9 ± 6.2**	**59.2 ± 7.48**	**4 M, 2 F**	**3 Ca, 3 Un**

*PMI* postmortem interval, *CTRY* young healthy individuals, *DS* young Down syndrome individuals, *DSAD* Down syndrome individuals affected by Alzheimer’s disease, *CTRO* old healthy individuals, *AA* African American, *Ca* Caucasian, *In* Indian, *Un* unknown

The ages of DS cases without AD were below 44 years, while the cases with both DS and AD were over 55 years. Likewise, neurotypical cases were split into two groups: controls young (CTRY, *n* = 6), to compare with the DS group, because they are ≤ 45 years; controls old (CTRO, *n* = 6), to compare with DSAD, because they are older than 45 years at death. Although the range of the age within young groups appears wide, it is quite similar between them, thus limiting the influence of age. This aspect agrees with previous studies both from our group and others [13, 15, 17–20]. The ANOVA analysis of postmortem interval (PMI) did not report significant differences among groups (*F* (3, 20) = 1.82; *p* = 0.1750). Similarly, no significant differences were observed in age between comparison groups (DS vs. CTR: *p* = 0.9926; DSAD vs. CTRO: *p* > 0.999). Sex was approximately evenly split in all the groups (four males and two females), except in the DSAD group (two males and four females). Groups selected in this study were chosen to maintain homogeneous age and gender. Proteomics data were analyzed by including PMI, age (where applicable), and sex as covariates in the statistical models.

### Mouse Samples

Ts66Yah mice were generated as previously described [21]. Animals were housed at up to four males and five females per cage (cage type: Green Line-39 × 20 × 16 cm, Techniplast, Italy) and had free access to purified water and food (D04 chow diet, Safe, Augy, France). The temperature in the animal house was maintained at 23 ± 1 °C, and the light cycle was controlled as 12 h light and 12 h dark (lights on at 7 AM). A total of 24 mice (*n* = 6 each group; three males and three females) were sacrificed by cervical dislocation at the same age, 3 and 9 months. Brain regions were immediately isolated after sacrifice and stored in dry ice for immunochemical experiments. The research project authorization was accredited by the French Ministry of National Higher Education and Research APAFIS#15187−201805221519333v3 granted to MRB and YH.

### Protein Sample Preparation

Human and mouse frontal cortices samples were homogenized in RIPA buffer (pH = 7.4) containing 50 mM Tris–HCl (pH = 7.4), 150 mM NaCl, 1% NP-40, 0.25% sodium deoxycholate,1 mM EDTA, 0.1% SDS, together with phosphatase and protease inhibitor (539132, Millipore, Burlington, MA, USA, 1:100; P0044; Sigma-Aldrich, St. Louis, MO, USA; 1:100). Samples were sonicated on ice and then centrifuged at 16,000 × rpm at 4 °C for 30 min to remove cellular debris. Supernatants were collected to determine total protein concentrations by the BCA method (Pierce, Rockford, IL, USA).

### Protein Expression Analysis by nLC–HDMSE

Protein extracts derived from both datasets (human and mouse samples) were handled for enzymatic digestion according to the filter-aided sample preparation (FASP) protocol with trypsin digestion (final trypsin concentration of 0.01 µg/µl) [22].

Label-free proteomic analysis was performed, as previously described by Greco V et al. with some modifications [23, 24]. First, 300 fmol/µL of digested enolase from *Saccharomyces cerevisiae* (P00924) was added to each sample as an internal standard. Each digested sample (0.25 µg) was loaded onto a Symmetry C18 5 µm, 180 µm 20 mm pre-column (Waters Corp., Milford, MA, USA) and was subsequently separated by a 120-min reversed-phase gradient at 300 nL/min (linear gradient, 2–40% ACN over 90 min) using a HSS T3 C18 1.8 µm, 75 µm 150 mm nanoscale LC column (Waters Corp., Milford, MA, USA) maintained at 40 °C. Tryptic peptides were separated on an ACQUITY MClass System (Waters Corp., Milford, MA, USA) and then separated peptides were analyzed using a high-definition Synapt G2-Si mass spectrometer (Waters Corp., Milford, MA, USA) directly coupled to the chromatographic system. Differential protein expression was evaluated by a high-definition expression configuration mode (HDMSE), a data-independent acquisition (DIA) protocol where ion mobility separation (IMS) is integrated into the LC-MSE workflow as described by Marini et al. [25]. The mass spectrometer parameters are set as follows: positive survey polarity of electrospray source (ES +), acquisition mode mass range 50–2000 m/z, capillary source voltage 3.2 kV, source T 80 °C, cone voltage 40 eV, TOF resolution power 20,000, precursor ion charge state 0.2–4, trap collision energy 4 eV, transfer collision energy 2 eV precursor MS scan time 0.5 s, and fragment MS/MS scan time 1.0 s. All spectra have been acquired in ion mobility separation mode (IMS) cycles with wave height at 40 V, wave velocity of 650 m/s, transfer wave height 4 V, and transfer wave velocity of 175 m/s. Data were post-acquisition lock mass corrected using the doubly charged monoisotopic ion of [Glu1]-Fibrinopeptide B (Waters Corp., Milford, MA, USA), sampled every 30 s. Each sample was run in three technical replicates. The analysis of differentially expressed proteins was performed according to Silva et al. [26] and Visser et al. [27].

Continuum LC–MS data from the three analytical replicates for each sample, derived from both human and mouse datasets, were processed for qualitative and quantitative analysis using the Protein Lynx Global Server v3.0.3 software (PLGS, Waters Corp., Milford, MA, USA). The qualitative identification of proteins was obtained using the embedded ion accounting algorithm of the software PLGS, and by searching against the *Homo sapiens* database (UniProt KB/Swiss-Prot Protein Knowledgebase restricted to homo sapiens taxonomy), and the *Mus musculus* database, respectively, to which the sequence from *Saccharomyces cerevisiae* Enolase (UniProtKB/Swiss-Prot AC: P00924) was appended. PLGS software search parameters were set as follows: automatic tolerance for precursor ions and product ions, a minimum of 1 fragment ion matched per peptide, a minimum of three fragment ions matched per protein, a minimum of two peptides matched per protein, two missed cleavages, carbamidomethylation of cysteines, and oxidation of methionine as fixed and variable modifications, respectively. Protein identification was based on the detection of more than two fragment ions per peptide and more than two peptides measured per protein. The false discovery rate (FDR) of the identification algorithm was set under 1%, based on a target-decoy database. For quantitative expression analysis, 300 fmol of Enolase has been set as the calibration protein concentration. PLGS software uses the most reproducible proteotypic peptides for retention time and intensity of Enolase digestion (m/z 745.43, m/z 814.49, m/z 1288.70, m/z 1416.72, m/z 1578.80, and m/z 1840.89) to normalize the table of the exact mass on retention times (EMRTs). The expression analysis was performed considering the experimental groups, including all the technical replicates from each sample, following the hypothesis that each group was an independent variable.

The differentially expressed proteins dataset was screened and filtered according to the following MS-established criteria by considering only those identifications from the alternate scanning LC-HDMSE data exhibiting a good replication rate (at least two out of three runs of the same sample) and with *p* < 0.05 for the relative protein fold change (two-tailed Student’s *t* test). The Benjamini–Hochberg correction was applied to the resulting *p*-values to control the false discovery rate (FDR), ensuring statistically robust identification of differentially expressed proteins in a high-throughput context. Furthermore, proteomics data were analyzed by including PMI, age (where applicable), and sex as covariates in the statistical models. Each of the six comparison groups was individually analyzed, and the significance of the regulation level was specified with a fold change of regulation higher than ± 30%, which is typically 2–3 times higher than the estimated error on the intensity measurement, used as a threshold to identify significant up or downregulation.

### Protein Ontologies and Network Analysis

To identify biologically relevant molecular pathways, the proteomic datasets were analyzed using bioinformatic tools based on the following databases and software analysis. The UNIPROT (the universal protein resource) database (https://www.uniprot.org) was employed to evaluate protein characteristics, including gene/protein name, length, sub-cellular localization, chromosomal distribution, and PTMs. QIAGEN’s Ingenuity Pathway Analysis (IPA) (http://www.qiagen.com/ingenuity) was used. For IPA software, the analysis parameters were set as follows: direct and indirect relationships, endogenous chemical substances included, all molecules and/or relationships considered the summary filter. The most significant pathways, categories, diseases, and functions associated with the loaded datasets were identified by calculating their significance (*p*-value, Fisher’s test). A *p*-value threshold was set at 0.05, which showed the probability of association between genes/proteins present in the datasets and each pathway/function (canonical pathway, biological function, Network interactions). STRING (Search Tool for the Retrieval of Interacting Genes/Proteins) software (https://string-db.org) was used to evaluate protein interaction and clustering. For STRING network analysis, the confidence level was set as medium (0.400), while the MCL clustering method was set with the inflation parameter of 3. Our goal in using multiple enrichment tools was to cross-validate key biological themes and increase the robustness of the functional interpretation, given the complementary strengths of each tool and database. This allowed exploring functional associations relevant to the experimental results from UNIPROT. Venn diagrams were built using EVenn software (http://www.ehbio.com/test/venn/) [28]. Additionally, data were analyzed and plotted by GraphPad Prism 10.4 software.

## Results

In this study, we used a label-free nLC–HDMSE proteomics approach on a total of 24 human frontal cortex samples divided into four groups (Table 1). To evaluate differentially expressed proteins (DEPs) in each group, identified proteins and their expression values have been matched in four different comparison sets considering genotype and aging as variables (DS vs. CTRY; DSAD vs. CTRO; DS vs. DSAD; CTRY vs. CTRO) (Sup. Table 2). Each comparison group was analyzed individually to assess specific variables or paired with others to evaluate changes associated with genotype or aging. In addition, we examined a novel mouse model of DS, Ts66Yah, alongside its euploid counterparts at 3 and 9 months of age, to investigate genotype-related alterations across age groups. These findings were then compared with those from young and aged human DS cohorts to identify conserved molecular mechanisms between species.

### Analysis of DEPs in Young DS and CTR Groups to Assess Early Proteome Alteration Associated with Genotype

**Fig. 1:**
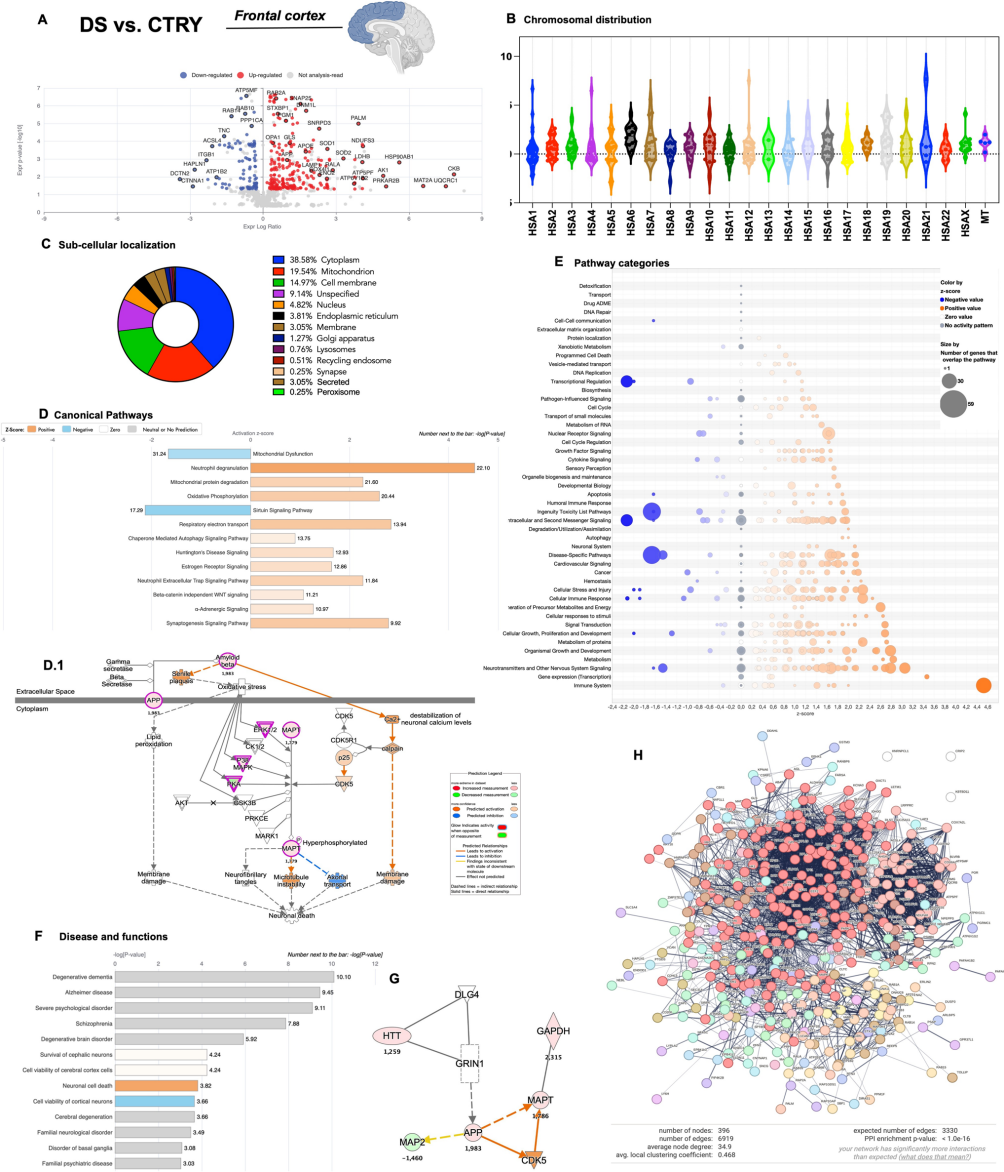
Genotype-associated protein expression analysis in young DS vs. CTR frontal cortices.** A** Volcano plot of DEPs between DS and CTRY samples. Red dots show significant (*p* < 0.05) DEPs with increased expression (positive log ratio), while blue dots show DEPs with decreased expression (negative log ratio). **B** Violin plot showing DEPs distribution among human chromosomes. **C** Analysis of DEPs subcellular localization. **D** Representative bar graph of canonical pathway analysis reporting significance (-log(*p*-value)) and activity pattern (*z*-score); the full list is reported in Sup. Table 5. **D.1** Canonical pathway describing DEPs associated with amyloid production and microtubule instability. **E** Bubble plot of pathway category analysis with category description, *z*-score, and significance (-log(*p*-value); bubble size). **F** Bar graph of downstream effects associated with DEPs and categorized as disease and functions (-log(*p*-value)); the full list is reported in Sup. Table 7. **G** Interaction network involving APP and tau overexpression. **H** Network interaction map by STRING analysis with n# of nodes, n# of degrees, avg. nodes degree, avg. local clustering coefficient, expected number of nodes, and PPI enrichment *p*-value

The comparison between DS and CTRY enabled the identification of DEPs attributable to genotype. This analysis facilitated the characterization of early molecular alterations in the brain associated with the triplication of HSA21. Our analysis brought the identification of 432 DEPs, as reported in the Volcano plot (Fig. 1A). Of those, 301 (69.6%) showed increased expression in the DS group, while 131 (30.4%) demonstrated decreased expression (Sup. Table 3). By interrogating the UNIPROT database, we evaluated the chromosomal distribution of genes coding for the identified DEPs (Fig. 1B and Sup. Table 4). We observed that ~ 2% of total DEPs were encoded on HSA21, while the majority of DEPs were attributable to HSA17 (7%), HSA11 (6.6%), and HSA12 (5.7%), supporting that HSA21 triplication had a considerable impact on disomic genes. Intriguingly, DEPs belonging to HSA21 included SOD1, S100B, APP, CBR1, ATP5PF, ATP5PO, and CCT8, and their expression levels were in line with previous DS brain studies [29]. The investigation of the DEPs’ subcellular localization established that 38.6% of DEPs were cytoplasmic proteins, while 19.5% were mitochondrial, 18% were membrane or cell-membrane associated proteins, 4.8% were nuclear proteins, and 3.8% of DEPs belonged to the endoplasmic reticulum (ER) (Fig. 1C.1) These results suggested an enrichment of mitochondrial brain protein modulated by genotype at a young age [30]. Of interest, 0.25% of DEPs were synaptic proteins. Remarkably, by comparing results from the young DS group with the database on DS frontal cortex from the Cancedda laboratory [31], we observed considerable overlap of DEPs (86.6% of our dataset) (Sup. Figure 4 and Sup. Table 15).

Subsequently, we used IPA software (Qiagen) to identify pathway categories, diseases and functions, and networks associated with DEPs in the young genotype group (Fig. 1D). We observed a significant negative *z*-score effect for mitochondrial dysfunction (−1.7), sirtuin signalling regulation (−2.1), and apoptosis signalling (−1.6), while a positive *z*-score effect was exerted by DEPs on energy-related pathways (oxidative phosphorylation + 2.6; respiratory electron transport + 2.8; glycolysis/gluconeogenesis + 1.13; AMPK signalling + 1.15), neurotransmission (synaptogenesis + 2.8; synaptic long-term potentiation/depression + 1.9), proteostasis (chaperone-mediated autophagy + 0.9; protein ubiquitination/deubiquitination + 1.3; unfolded protein response (UPR) + 1.3; mTOR signalling + 1.9; and EIF2 signalling + 1.7), NRF2-mediated response (+ 1.9) and JAK/STAT signalling (+ 1.6) (Sup. Table 5). Furthermore, several DEPs were associated with amyloid fiber formation (+ 2.2) (e.g., APP, ERK1,2, p38 MAPK, APOE) (Fig. 1D.1). Of interest, our data position APOE alteration as an early molecular event in individuals with DS (+ 1.7-log fold), supporting its role in brain alteration and the transition to AD pathology. Accordingly, recent evidence suggests that APOE overexpression may influence the development of pathological hallmarks in the brains of individuals with DS, thereby modulating disease mechanisms with potential clinical implications [31, 32]. Pathway categories analysis confirmed that the positive fold change of DEPs in the DS vs. CTRY comparison resulted in an increased positive *z*-score effect across most pathways (Fig. 1F). The main pathway categories affected by genotype included neurotransmitter and nervous system signalling, generation of precursor metabolites and energy, as well as disease-specific pathways. Disease and function analysis by IPA supported a role for DEPs in the development of neurological diseases associated with degenerative dementia, AD, psychological disorders, and developmental disorders (Fig. 1G and Sup. Table 7). Intriguingly, network analysis highlighted mechanisms promoting APP and MAPT (tau) intersection in the young DS group that might promote pathological hallmarks formation (Fig. 1G and Sup. Table 6). By evaluating DEPs from young DS with the NeuroPro database from AD and early stages studies (https://neuropro.biomedical.hosting), as described by Askenazi et al. [33], we observed a robust overlap with DEPs detected in the frontal cortex of AD patients, particularly those showing consistent plaque formation (Sup. Fig. 5 and Sup. Table 16). We then performed STRING software analysis to evaluate the DEPs interaction and network association (Fig. 1H). STRING data reported 396 nodes and 6919 edges, with an average node degree of 34.9, an average local clustering coefficient of 0.468, and a PPI enrichment *p*-value < 1.0 e−16 (Sup. Table 9). The cluster analysis reported 51 different clusters that include carboxylic acid metabolic process (159 DEPs), oxidative phosphorylation (36 DEPs), proteasome (15 DEPs), and serotonin neurotransmitter release cycle (12 DEPs), among others (Sup. Fig. 2 and Sup. Table 8), thus supporting the alteration of bioenergetic pathways, proteostasis mechanisms, and neurotransmission in young DS individuals.

**Fig. 2:**
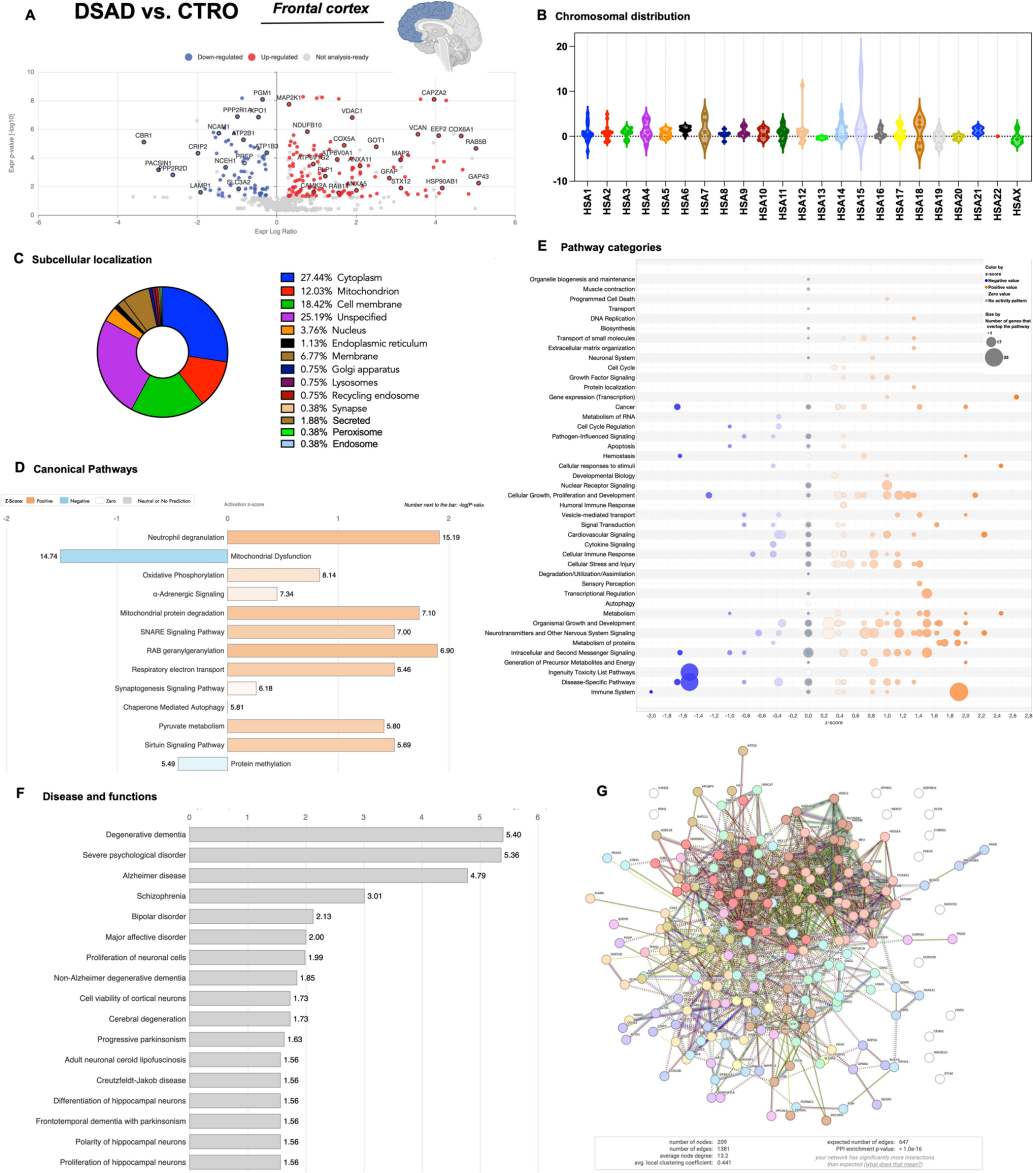
Genotype-associated protein expression analysis in DSAD vs. CTRO frontal cortices.** A** Volcano plot of DEPs between DSAD and CTRO samples. Red dots show significant (*p* < 0.05) DEPs with increased expression (positive log ratio), while blue dots show DEPs with decreased expression (negative log ratio). **B** Violin plot showing DEPs distribution among human chromosomes. **C** Analysis of DEPs sub-cellular localization. **D** Representative bar graph of canonical pathway analysis reporting significance (-log(*p*-value)) and activity pattern (*z*-score); the full list is reported in Sup. Table 5. **E** Bubble plot of pathway category analysis with category description, *z*-score, and significance (-log(*p*-value); bubble size). **F** Bar graph of downstream effects associated with DEPs and categorized as disease and functions (-log(*p*-value)); the full list is reported in Sup. Table 7. **G** Network interaction map by STRING analysis with n# of nodes, n# of degrees, and avg. nodes degree, avg. local clustering coefficient, expected number of nodes, and PPI enrichment *p*-value

### Analysis of Cortical Differences in Aged DS (Affected by AD) and CTR Groups to Assess Late Proteome Alteration Associated with Genotype and AD Development

The analysis of DEPs in individuals with DS and AD (DSAD) compared to aged controls (CTRO) enabled us to investigate and validate the impact of genotype in older age while accounting for the influence of Alzheimer’s pathology. The proteome evaluation of the DSAD vs. CTRO comparison group identified 281 DEPs (Sup. Table 2), of which 190 (67.6%) were increased, while 91 (32.4%) were decreased following a trend similar to what was observed in younger individuals (Fig. 2A and Sup. Table 3). The chromosomal distribution of DEPs coding gene confirmed the relatively minor involvement of HSA21 (1% of total DEPs), while HSA1 (6%), HSA11 (5%), and HSA17 (4.2%) showed major alterations (Fig. 2B). Once more, our data supported a role for HSA21 triplication in driving the alteration of disomic genes. Focusing on DEPs’ subcellular localization, we observed that 27.4% were in the cytoplasm, 25.1% in the membrane, while 12% were mitochondrial proteins (Fig. 2C.1). In the comparison of old controls and DSAD groups, we observed a lower implication of mitochondrial-resident DEPs but a higher involvement of membrane proteins. Furthermore, synaptic proteins represented 0.4% of total DEPs. The analysis of canonical pathways by IPA confirmed the negative *z*-score effect on mitochondrial dysfunction (−1.6), but also for NRF2 response (−0.38) and insulin receptor signalling (−0.8), among others. Instead, a positive *z*-score effect was identified for energy-related pathways (TCA cycle + 2; glycolysis + 2; AMPK signalling + 1), neurotransmission (synaptogenesis + 0.5; synaptic long-term potentiation/depression + 1.1), and proteostasis (chaperone-mediated autophagy + 1.06; autophagy + 1.13) (Fig. 2D and Sup. Table 5). Intriguingly, the observed induction of autophagy pathways aligns with previous findings in DS, which reported activation of the early phases of autophagy but also inhibition of later stages [15, 34]. The analysis of pathway categories also supported a dominance of positive *z*-score pathways associated with increased DEPs, but it was less pronounced in comparison to the DS group (Fig. 2F). The analysis of downstream effects identifies DEPs with roles in promoting neurological disorders, among which are listed AD, psychological, and developmental disorders (Fig. 2G and Sup. Table 7). By comparing DEPs from DSAD vs. CTRO comparison with the NeuroPro databases of AD studies [33], a strong overlap (about 62%) was noticed with proteome changes of AD, as well as with Aβ plaques (Sup. Figure 5 and Sup. Table 17). In addition, functional alterations exerted by DEPs involved cellular growth and development, cell morphology, and cell death and survival (Fig. 2G and Sup. Table 7). The STRING network analysis reported 209 nodes with 1381 edges (with respect to 647 expected), an average node degree of 13.2, an average local clustering coefficient of 0.44, and PPI enrichment *p*-value < 1.0e−16 (Fig. 2E.1 and Sup. Table 9). Primary clusters identified by the software included protein folding chaperone (19 DEPs) and oxidative phosphorylation (18 DEPs), among others (Fig. 2E.2 and Sup. Table 8).

### Genotype-Related Protein Changes Common to Young (DS vs. CTRY) and Old (DSAD vs. CTRO) Comparison Groups

**Fig. 3:**
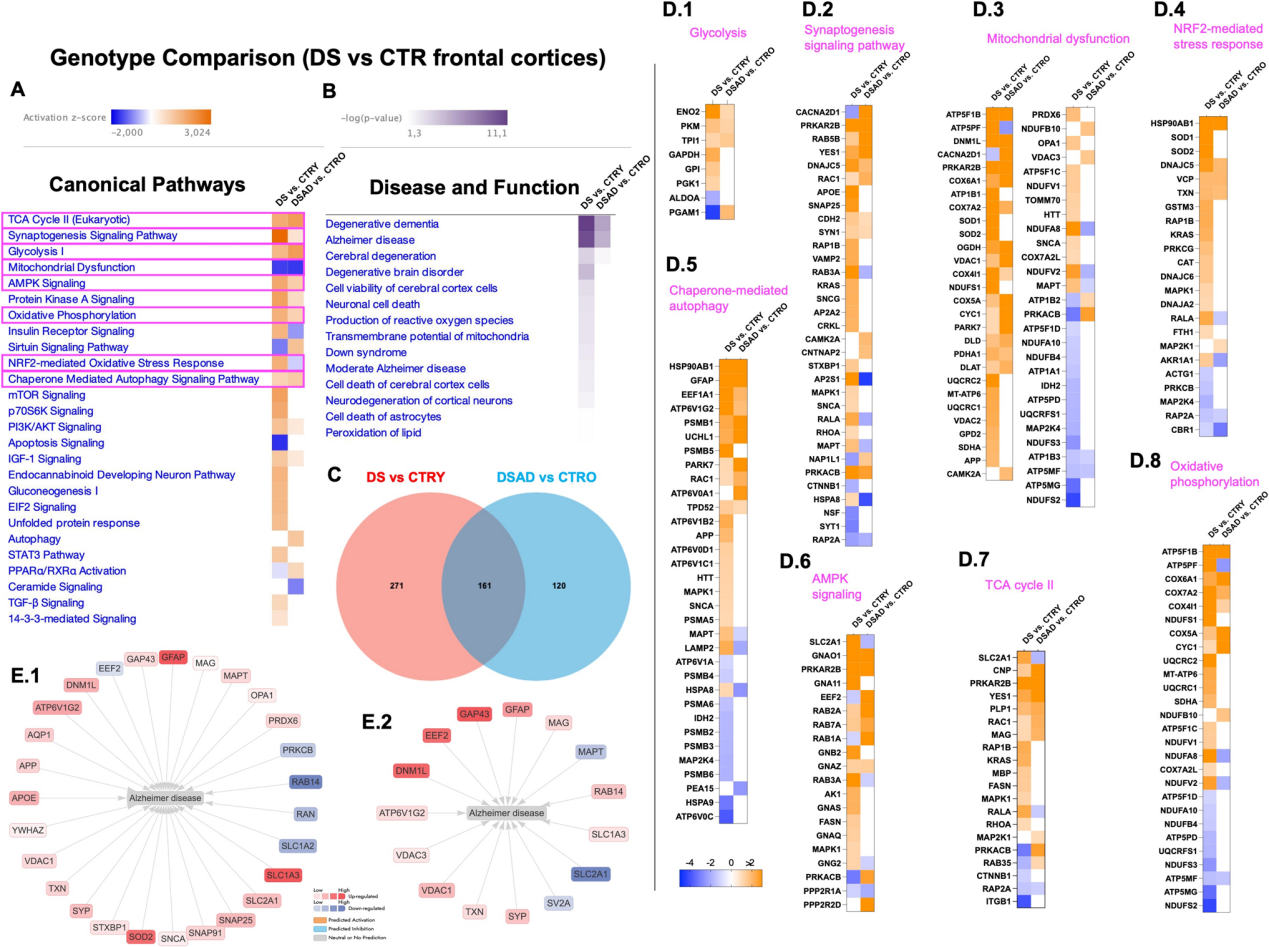
Comparison between genotype-associated analyses in young and old DS vs. CTR groups. **A** Heat map of canonical pathway comparison between DS vs. CTRY and DSAD vs. CTRO groups reporting *z*-score activation. Overlapping pathways are highlighted in pink. **B** Heat map of diseases and functions associated with DEPs in DS vs. CTRY and DSAD vs. CTRO groups (-log(*p*-value)). **C** Venn diagram of overlapping DEPs between young and old genotype comparison groups. **D** DEPs heat map for glycolysis (**D.1**), synaptogenesis signalling pathway (**D.2**), mitochondrial dysfunction (**D.3**), NRF2-mediated stress response (**D.4**), chaperone-mediated autophagy (**D.5**), AMPK signalling (**D.6**), TCA cycle II (**D.7**), and oxidative phosphorylation (**D.8**). **E** Protein associated with AD development in DS (**E.1**) and in DSAD (**E.2**) individuals, in red overexpressed proteins and blue underexpressed proteins

To further investigate the effect of genotype in young and old DS individuals and the involvement of AD development, we performed a genotype-specific analysis between the two comparison groups described above (DS vs. CTRY and DSAD vs. CTRO). The Venn diagram shows 161 DEPs shared by both groups, 271 DEPs unique to the DS vs. CTRY group and 120 DEPs unique to the DSAD vs. CTRO group (Fig. 3C). The canonical pathway analysis allowed us to recognize the *z*-score trend and DEPs components common between the comparison groups (Fig. 3A). Intriguingly, several pathways followed the same *z*-score trend of induction/repression and reported the overlap of several DEPs components, including glycolysis (ENO2, PKM, TPI1), mitochondrial dysfunction (ATP5F1B, DNM1L, PRKAR2B, COX6A1, VDAC1, PDHA1, PARK7, CYC1), chaperone-mediated autophagy (HSP90AB1, GFAP, UCHL1, TPD52, EEF1A1), TCA cycle II (CNP, MAG, PLP1, RAC1, YES1), and oxidative phosphorylation (COX5A, COX7A2, COX4I1, CYC1) (Fig. 3D.1,3,5,7,8). This trend supported the occurrence of pathological mechanisms associated with genotype but independent of aging or AD development. However, an opposite *z*-score trend was observed for NRF2 redox response (Fig. 3D.4), which declined in DSAD vs. CTRO groups with respect to DS vs. CTRY, supporting a functional switch in old DS individuals associated with age and AD progression (Sup. Table 11). NRF2-associated proteins with differential expression between young and old groups included RALA, AKR1A1, and MAP2K1 (Fig. 3D.4). The analysis of DEPs downstream effects on disease and functions further suggested their common involvement in promoting degenerative dementia and AD (Fig. 3B). The evaluation of proteins associated with AD development in young DS individuals and in DSAD comparison with age-matched controls supported the shared alteration of GFAP, SLC1A3, DNM1L, VDAC1-3, GAP43, MAG, SYP, ATP6V1G2, and TXN, among others (Fig. 3E.1,2). Intriguingly, the effect on AD-related proteins appeared more pronounced in young individuals with DS compared to those with DSAD, where the pathology is already established, suggesting that molecular changes associated with HSA21 triplications could precede the overt manifestation of AD pathology [18].

### Analysis of Aging and AD Signatures in Young and Old DS Individuals (DSAD vs. DS)

**Fig. 4:**
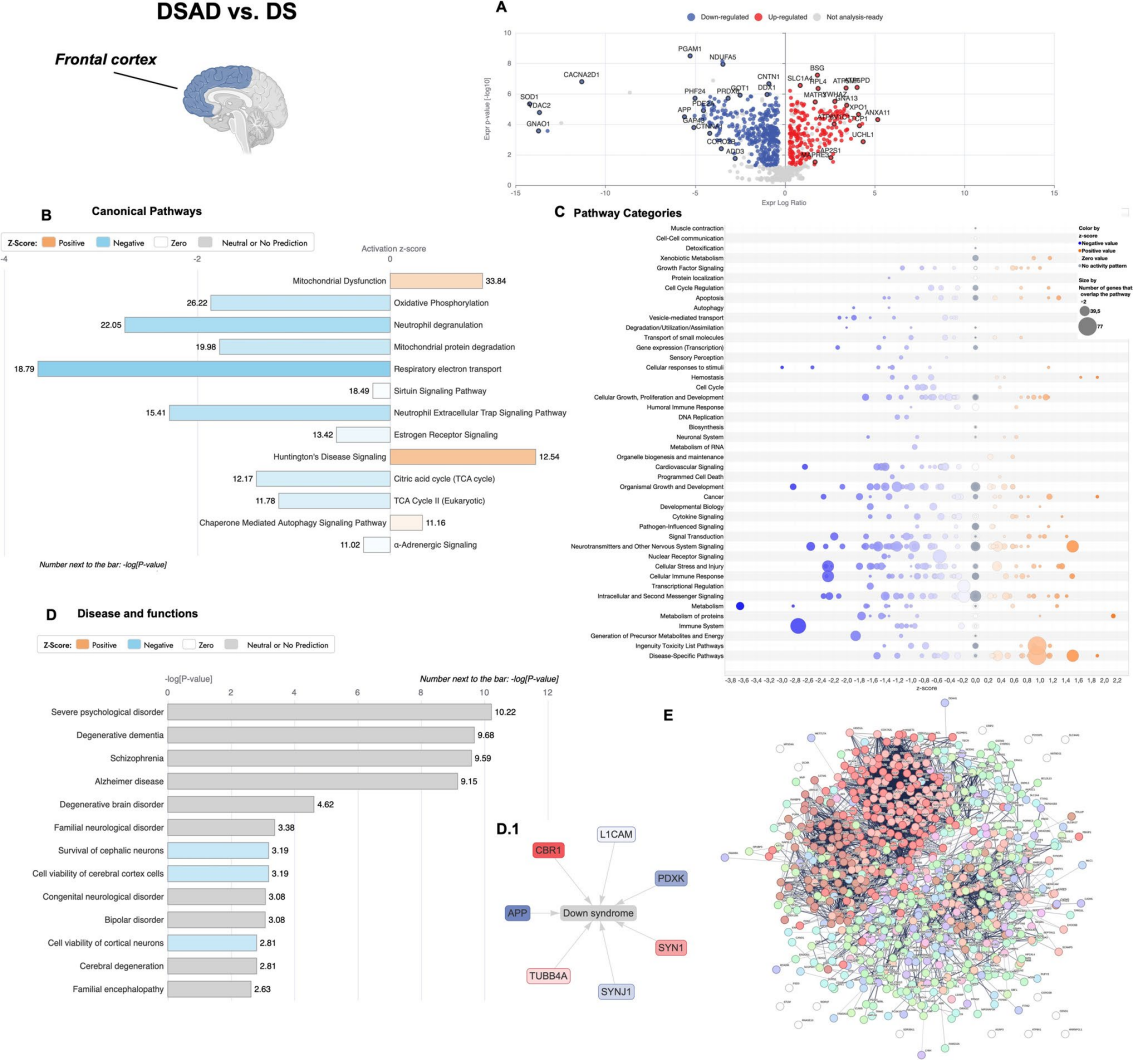
Aging-associated protein expression analysis in young and old DS human cortices (DSAD vs. DS). **A** Volcano plot of DEPs in the DSAD vs. DS comparison group. Red dots show significant (*p* < 0.05) DEPs with increased expression (positive log ratio), while blue dots show DEPs with decreased expression (negative log ratio). **B** Representative bar graph of canonical pathway analysis reporting significance (-log(*p*-value)) and activity pattern (*z*-score); the full list is reported in Sup. Table 5. **C** Bubble plot of pathway category analysis with pathway description, *z*-score, and significance (-log(*p*-value); bubble size. **D** Representative bar graph of downstream effects associated with DEPs and categorized as disease and functions (-log(*p*-value)); the full list is reported in Sup. Table 7. **D.1** DEPs associated with DS pathology from disease and function analysis. **E** Network interaction map by STRING analysis with n# of nodes, n# of degrees, and avg. node degree, avg. local clustering coefficient, expected number of nodes, and PPI enrichment *p*-value

**Fig. 5:**
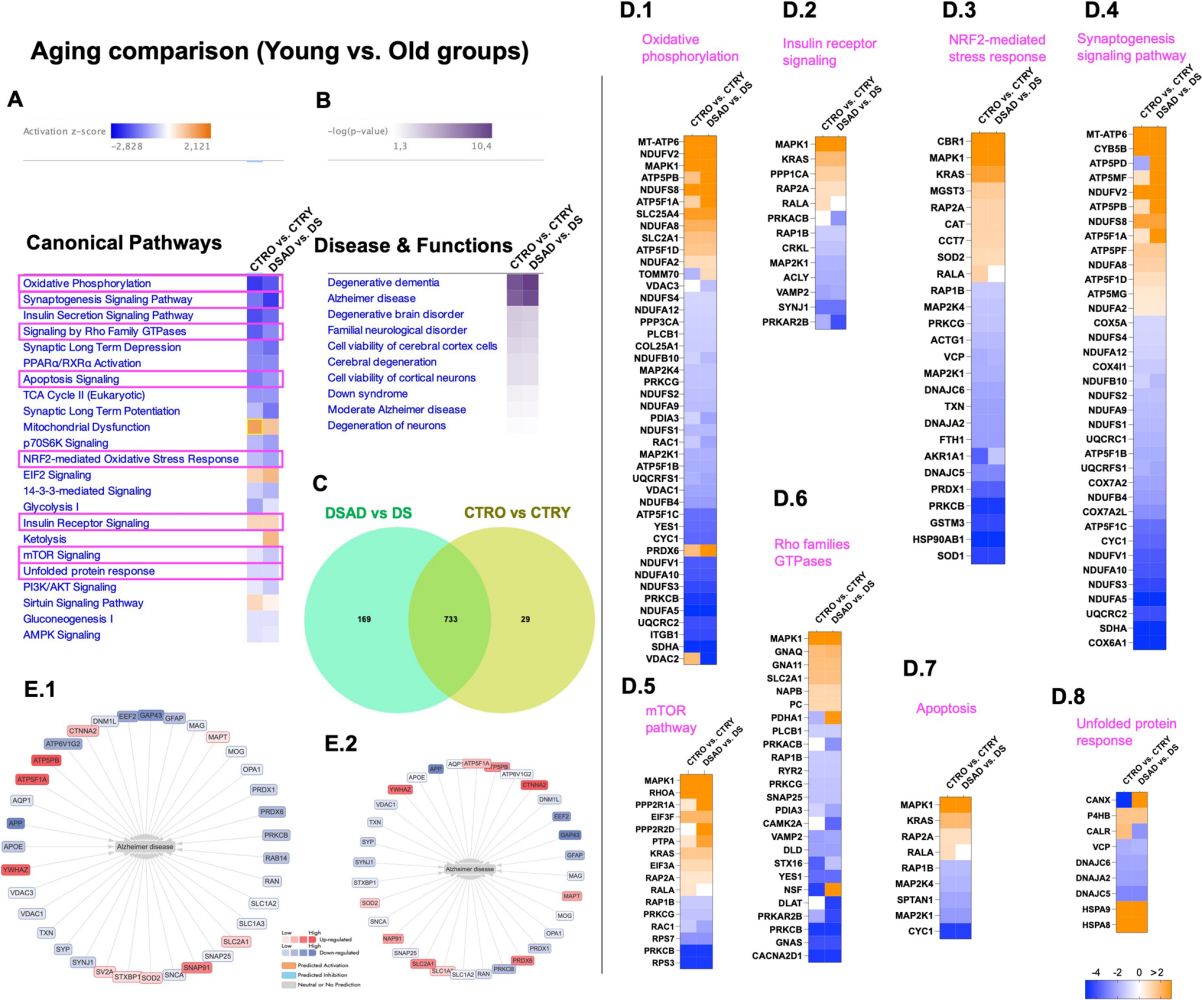
Comparison between age-associated analyses in the DS and CTRs groups.** A** Heat map of canonical pathway comparison between DSAD vs. DS and CTRO vs. CTRY groups reporting *z*-score activation. Overlapping pathways are highlighted in pink. **B** Heat map of diseases and functions associated with DEPs in DSAD vs. DS and CTRO vs. CTRY groups (-log(*p*-value)). **C** Venn diagram of overlapping DEPs between DS and CTR aging comparison groups. **D** Heat map of DEPs involved in oxidative phosphorylation (**D.1**), insulin receptor signalling (**D.2**), NRF2-mediated stress response (**D.3**), synaptogenesis signalling pathway (**D.4**), mTOR pathway (**D.5**), Rho family GTPases (**D.6**), apoptosis (**D.7**), unfolded protein response (**D.8**). **E** Protein associated with AD development as effect of aging in DS individuals (DSAD vs. DS; **E.1**) and in CTRs (CTRO vs. CTRY, **E.2**), in red overexpressed proteins and blue underexpressed proteins

Secondary to genotype, we aimed to assess the effect of aging and AD development in promoting the increased/decreased DEPs in the experimental groups sharing DS pathology (DSAD vs. DS). The comparison of DSAD vs. DS groups identified 902 DEPs, of which 336 (37%) were increased and 566 (63%) were decreased in aged DS individuals with AD (Fig. 5A and Sup. Table 3). Intriguingly, proteomics analysis supported a generalized decline of protein expression in the DS cerebral cortex as a result of increased age and AD progression. The IPA canonical pathways analysis demonstrated a positive *z*-score for mitochondrial dysfunction (+ 0.8), JAK/STAT signalling (+ 1.1), EIF2 signalling (+ 0.9), and apoptotic execution phase (+ 0.38), while a negative *z*-score was evidenced for energy-related pathways (oxidative phosphorylation −1.85; respiratory electron transport (−3.65), TCA cycle −1.15; AMPK signalling −0.27), NRF2 redox response (−0.77), ERK/MAPK pathway (−2.35), chaperone-mediated autophagy (−1.89), CREB signalling (−1.6), mTOR signalling (−0.3), and mitophagy (−1), among others (Fig. 5B and Sup. Table 5). Remarkably, we observed a negative *z*-score for the amyloid fiber formation pathway (−1.63) with expression for APP, H2AX, MFGE8, APOE, SNCA, and GSN in DSAD cortex compared to young DS. These findings suggest that individuals with DS experience early activation of mechanisms contributing to the development and deposition of pathological signatures. In DSAD, these mechanisms are further modulated by aging and AD progression, rather than by genotype alone (Fig. 4B and Sup. Table 5). The pathway category analysis reported in the DSAD vs. DS comparison showed a predominant negative *z*-score effect on pathways involving neurotransmitters and nervous system signalling, cell stress and injury, and organismal/cell growth and development (Fig. 4C). As previously observed for genotype, age-related changes of DEPs might contribute to neurological diseases, including AD, psychological, metabolic, and developmental disorders (Fig. 4D and Sup. Table 7), by interfering with crucial cellular functions including cell death and survival, cell growth, DNA replication, recombination, and repair. The network analysis showed the interaction of DEPs strongly involved in carbon metabolism (108 DEPs) and proton motive force-driven mitochondrial ATP synthesis (57 DEPs) (Fig. 4E and Sup. Table 8).

### Analysis of Aging Signatures in CTR Groups

We further compared young and old neurotypical control groups (CTRO vs. CTRY) to evaluate the effect of aging in the absence of DS and AD. The analysis of the CTRO vs. CTRY comparison group identified 760 DEPs; 261 (34.3%) were increased, and 499 (65.7%) were decreased (Sup. Figure 1 and Sup. Table 3). A similar trend in protein expression was observed in the age group comparisons relative to DSAD vs. DS. Canonical pathways confirmed the effect of aging in inducing mitochondrial dysfunction (+ 0.8 *z*-score), JAK/STAT signalling (+ 1.1 *z*-score), EIF2 signalling (+ 0.3 *z*-score) (Fig. 5F), while a negative *z*-score effect was observed for neurotransmission (neurotransmitter release cycle −1.9 *z*-score) and energy-related pathways (oxidative phosphorylation −1.5 *z*-score), as also confirmed by pathways category analysis (Sup. Figure 1B, C and Sup. Table 5). As well, the effect of aging in CTR groups confirmed a role for DEPs in promoting the development of pathological outcomes, such as the alteration of cell death and survival, and of DNA replication, recombination, and repair that are not only attributable to the DS phenotype (Fig. 5H and Sup. Table 7). Notably, the analysis of age-related DEP changes in CTR cortices supported the initiation of processes promoting the formation and the quantity of amyloid fibrils (Sup. Figure 1F) due to the expression changes for APOE, APP, SYNPO, SYNJ1, SOD2, and CYP46A1. This effect is comparable to the one observed in young individuals with DS, further supporting the view of DS as a condition characterized by premature aging [32, 35].

### Age-Related Protein Changes Common to DS (DSAD vs. DS) and CTRs (CTRO vs. CTRY) Comparison Groups

The evaluation of the DS and CTRs comparison group enabled us to delineate molecular processes associated with aging, independent of DS or AD pathology. We observed overall that in the old vs. young groups, about 80% of DEPs overlapped (Fig. 5C). Perhaps unsurprisingly, this overlap in DEPs revealed several altered pathways which were also observed in the canonical pathway analyses (Fig. 5A). These similarities include the negative regulatory trend on aging and components of metabolic pathways (oxidative phosphorylation D.1; mitochondrial dysfunction D.2), proteostasis network (mTOR signalling D.6; UPR D.9), NRF2-mediated redox response (D.3), and synaptogenesis signalling pathway (D.5), among others (Sup. Tables 11 and 12). However, as expected, the aging effect appeared more severe in the DS comparison group than that observed in the CTR groups. Remarkably, DEPs overlapping between DS and CTRs that were associated with aging and AD development included MAPT, ATP5PB, SNAP91, SOD2, YWHAX, GAP43, EEF, SYNJ1, and OPA1, among others (Fig. 5E.1,2).

### Analysis of Cortical Differences in Old and Young DS and CTR Groups to Assess Common and Divergent Effects on the Proteome Associated with Genotype and Aging

**Fig. 6:**
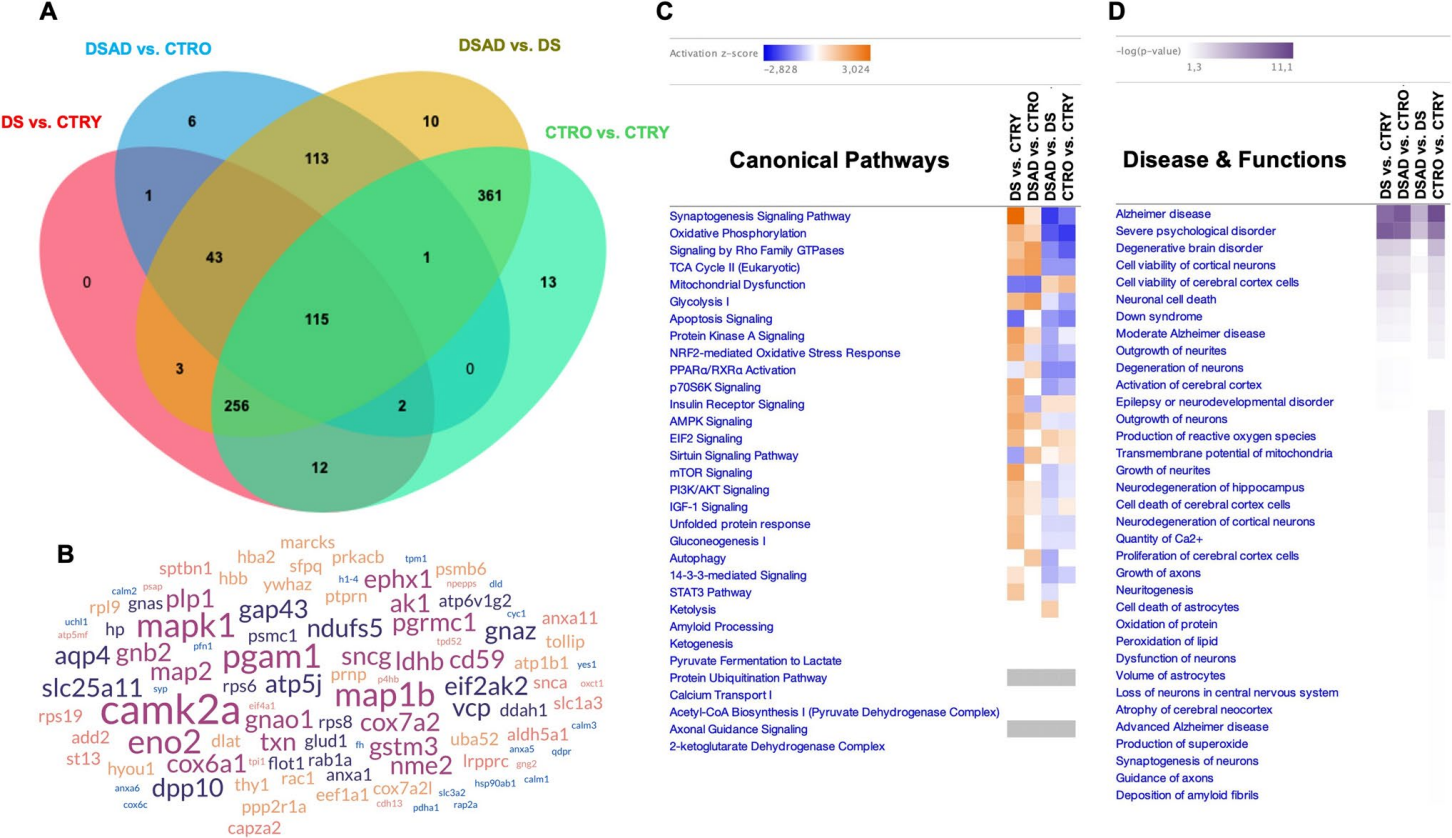
Comparison between genotype and aging-associated groups.** A** Venn diagram of overlapping DEPs between DS and CTR groups analyzed for genotype and aging effect. **B** World cloud showing proteins and their isoforms common to different groups of comparison. **C** Heat map of canonical pathway comparison between DS and CTRs groups reporting *z*-score activation. Overlapping pathways are highlighted in pink. **D** Heat map of disease and functions associated with DEPs in DS and CTR groups (-log(*p*-value))

The evaluation of overlapping DEPs between the four comparison groups (genotype + aging) demonstrated that 115 (12.3%) DEPs were identified across analyses and were modified by both genotype and aging. The world cloud analysis allowed for highlighting the DEPs commonly identified by proteomics. Intriguingly, the four groups of comparison reported the common modulation (either increase or decrease) for CAMK2A, MAP1B, PGAM1, ENO2, MAPK1, GAP43, NDUFS5, EIF2AK2, DPP10, SLC25A11, AQP4, VCP, GNAZ, among others. These proteins and their isoforms resulted in a high susceptibility to expression changes associated with HSA21 triplication and/or aging. However, the analysis of canonical pathways revealed that the impact of DEPs on brain molecular mechanisms was predominantly driven by either genotype or aging, with these factors exhibiting opposing effects. Specifically, genotype tended to enhance pathway activation, whereas aging primarily exerted a suppressive influence (Fig. 6B). The interplay of these factors resulted in modulation of energy-related pathways, the proteostasis network, and neurotransmission-related signaling in both DS and control groups (Sup. Table 14). Notably, the analysis of downstream effects across the four comparison groups indicated that alterations in DEPs contributed jointly to the development of AD (Fig. 6C). These findings suggest a parallel and reciprocal interaction between genotype and aging in the initiation of DS neuropathology.

### Genotype Differences in 3- and 9-Month-Old Ts66Yah Mice Compared to Euploids and Human DS to Evaluate Conserved Pathological Mechanisms

**Fig. 7:**
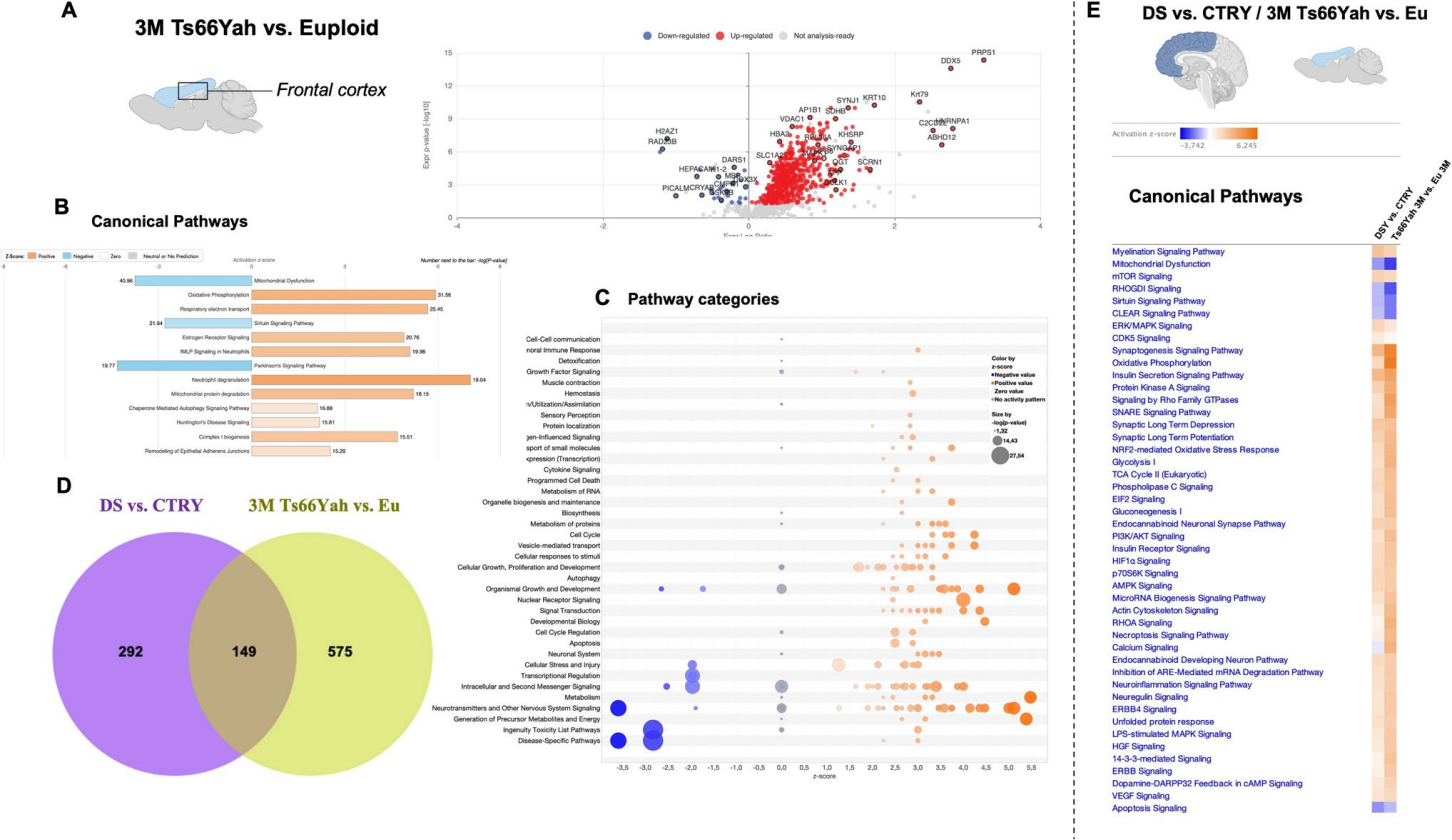
Genotype analysis of 3-month-old Ts66Yah and Euploids mice.** A** Volcano plot of DEPs between 3-month-old Ts66Yah vs. euploids (Eu) mice. Red dots show significant (*p* < 0.05) DEPs with increased expression (positive log ratio), while blue dots show DEPs with decreased expression (negative log ratio). **B** Representative bar graph of canonical pathway analysis reporting significance (-log(*p*-value)) and activity pattern (*z*-score), full list of canonical pathways in Sup. Table 13. **C** Bubble plot of pathway category analysis with category description, *z*-score, and significance (-log(*p*-value); bubble size). **D** Venn diagram of overlapping DEPs between DS vs. CTRY and 3-month Ts66Yah vs. Eu groups. **E** Heat map of canonical pathway comparison between DS vs. CTRY and 3-month Ts66Yah vs. Eu groups reporting *z*-score activation

We subsequently evaluated the novel murine model of DS, the Ts66Yah, at 3 and 9 months of age compared with euploid (Eu) mice (*n* = 6 per group; three males and three females) to further decipher the effects of trisomy on protein expression remodelling. As shown by us and others [21, 36], the Ts66Yah closely mimics behavioral, anatomical, and molecular changes observed in trisomy 21, thus representing a valuable murine model for DS research. No significant differences were observed for sex in Ts66Yah mice [36]. Our label-free nLC-HDMS^E^ analysis of frontal cortices in young (3 months of age) animals identified 724 DEPs between Ts66Yah and Eu mice (Fig. 7A); 687 DEPs (95%) were increased, while 37 (5%) were downregulated (Sup. Table 3). The analysis of canonical pathways and of pathway categories reported the increased *z*-score for energy-related pathways (oxidative phosphorylation + 5.3; glycolysis + 3.1 and glucose metabolism), stress responses (NRF2-mediated Oxidative Stress Response + 3; autophagy + 2.4), and neurotransmission (neurotransmitter release cycle + 3.4) (Fig. 7B, C, and Sup. Table 5). A decreased *z*-score was evidenced for mitochondrial dysfunction (−2.8) and sirtuin signalling pathways (−1.9) (Fig. 7B, C, and Sup. Table 5). By comparing young Ts66Yah mice with young DS cases, we observed an overlap of 149 (21.9%) DEPs (Fig. 7B). Similarities between mice and humans on the alteration of pathological mechanisms were corroborated by the comparison analysis between humans and mice data (Fig. 7E). This reported a superimposable effect of genotype on canonical pathways that coincided for *z*-score, pathway composition, and component expression trend (Sup. Table 13). Notably, our findings revealed a strong conservation of DS genotype-associated pathological mechanisms across humans and mice at a young age.

**Fig. 8:**
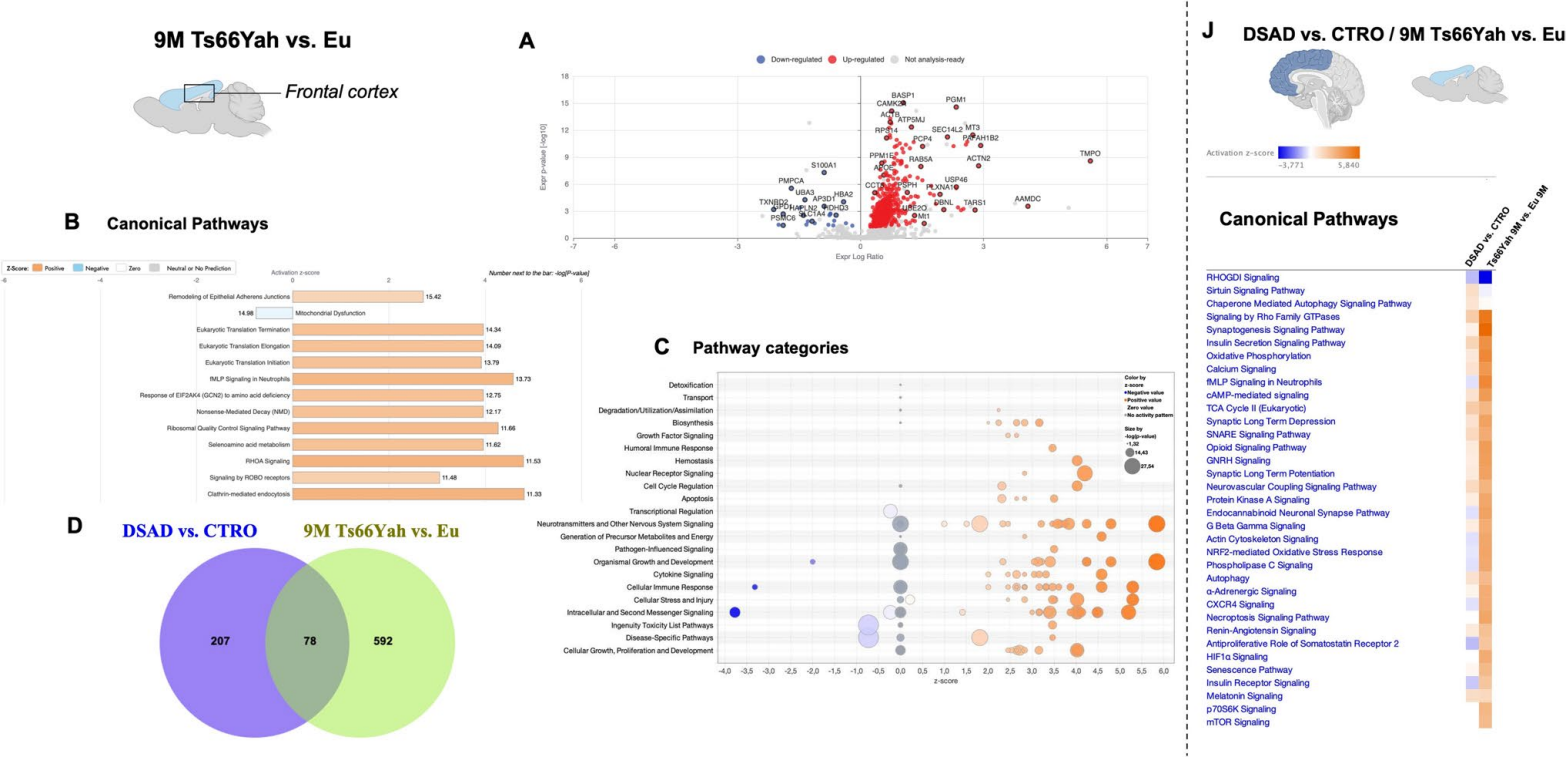
Genotype analysis of 9-month-old Ts66Yah and euploid mice.** A** Volcano plot of DEPs between 9-month-old Ts66Yah vs. Eu mice. Red dots show significant (*p* < 0.05) DEPs with increased expression (positive log ratio), while blue dots show DEPs with decreased expression (negative log ratio). **B** Representative bar graph of canonical pathway analysis reporting significance (-log(*p*-value)) and activity pattern (*z*-score), with a full list of canonical pathways in Sup. Table 13. **C** Bubble plot of pathway category analysis with category description, *z*-score, and significance (-log(*p*-value); bubble size). **D** Venn diagram of overlapping DEPs between DSAD vs. CTRO and 9-month Ts66Yah vs. Eu groups. **E** Heat map of canonical pathway comparison between DSAD vs. CTRO and 9-month Ts66Yah vs. Eu groups reporting *z*-score activation

The analysis of Ts66Yah at 9 months of age compared with Eu confirmed similarities in the effect of genotype between humans and mice, but also revealed discrepancies most likely associated with aging and/or AD development in humans. We identified in older mice 670 DEPs, with 637 (95%) increased and 33 (5%) decreased, thus maintaining the same distribution observed in young animals (Fig. 8A and Sup. Table 3). Protein overlaps between humans and mice at old age were less pronounced than in the young DS humans and mouse comparison, with only 78 (9.3%) orthologous proteins (Fig. 8D and Sup. Table 13). Reduced similarities between DSAD and 9-month Ts66Yah mice were confirmed by the comparison of canonical pathway and pathway categories, which show a similar *z*-score trend for mitochondrial dysfunction and neurotransmission-related pathways, but also discrepancies for NRF2 redox response (+ 3.4) and insulin receptor signalling (+ 2.2), among others (Fig. 8B, C, J and Sup. Table 5).

Collectively, the murine proteome showed a robust overlap with human data that addresses the genotype-dependent modulation of protein expression levels; however, the age-related phenotype of Tg mice partially diverges from the human proteome signature, most likely due to the lack of a severe AD phenotype [36].

## Discussion

The substantial variability and complexity of phenotypes associated with DS pose significant challenges to understanding the underlying biology. Comprehensive profiling of DS brains, including gene, transcript, and protein expression, enables the identification of dysfunctional pathways and co-expression networks. These approaches help uncover key drivers of age-related disease mechanisms and may advance the discovery of therapeutic targets. Molecular phenotypes in DS are shaped both by genomic instability and the presence of a third copy of HSA21. However, studies often report weak correlations between transcriptomic and proteomic data [11], highlighting the complexity of post-transcriptional regulation. Our findings, consistent with previous studies, indicate that approximately 90% of differentially expressed genes (DEGs) and DEPs in DS are located on disomic chromosomes. This suggests genome-wide dysregulation of gene expression, not limited to the triplicated region. The Espinosa laboratory [12] reported both overexpression of trisomic genes and marked inter-individual variability in expression patterns. While genomic studies elucidate the primary effects of HSA21 triplication, downstream impacts on transcription and translation across the genome remain poorly understood. Our study presents the first proteome-wide signature of the human DS brain, aiming to characterize variation in protein expression not only as a function of genotype but also with aging and AD pathology to provide a temporal pattern of biological alterations. Aebersold and colleagues (2017) demonstrated a weak correlation between DEGs and DEPs, further complicated by altered protein turnover in trisomic fibroblasts [37]. More recently, Rastogi et al. (2024) performed multi-omics profiling of the DS hippocampus and cortex, identifying significant dysregulation of genes, transcripts, proteins, and miRNAs [31]. Notably, non-triplicated genes were broadly affected, suggesting widespread genome-level consequences. Our proteomic analysis of postmortem frontal cortices revealed elevated protein levels in approximately 68% of DEPs across DS groups compared to neurotypical controls. However, while the ratio of upregulated to downregulated DEPs was consistent across DS groups, the total number of DEPs decreased with age and AD onset, suggesting an overall attenuation of protein expression. A similar age-dependent effect was observed in controls. Although HSA21 encodes only 247 protein-coding genes (~ 1.2% of the genome), just ~ 2% of DEPs in our dataset mapped to HSA21, highlighting the broader genomic impact. Canonical pathway analysis comparing DS and control groups revealed positive *z*-scores for pathways involved in energy metabolism, proteostasis, and redox responses. These changes likely reflect both direct effects from overexpression of HSA21 genes such as *ATP5PO*, *S100B*, *CBR1*, and *SOD1*, and secondary genome-wide regulatory effects involving disomic genes. Remarkably, we observed strong concordance between our DS frontal cortex proteome data and findings from Rastogi et al. [31], confirming consistency in protein alterations associated with trisomy 21. In addition, the finding of elevated levels of APOE, a well-recognized AD-associated protein, suggests that proteome changes outside HSA21 contribute throughout life to modulate the neuropathological phenotype most likely impinging on dementia onset. A similar observation was reported by Wiseman’s group, which proposed a role for APOE overexpression in the clinical manifestation of AD pathology in DS [32].

Loss of proteostasis is one of the most striking cellular consequences of trisomy 21 [38]. Aebersold et al. reported a mild but widespread increase in protein turnover in trisomic cells, with proteins from heteromeric complexes on HSA21 buffered by accelerated degradation [39]. This buffering limits the protein-level effects of gene dosage increases. In contrast, proteins encoded outside HSA21 show more variable responses, being increased, decreased, or unchanged, reflecting a more complex regulatory landscape. Our findings indicate that DS-related genotype changes disrupt cellular pathways essential for brain development and maturation, likely contributing to premature aging and increased co-morbidities. APP overexpression and altered amyloid processing were prominent, consistent with earlier analyses of Aβ deposition in DS brains by Cenini et al. [13]. Our IPA network analysis revealed APP–tau interactions in DS brains, suggesting that their toxic synergy occurs well before clinical dementia onset. These mechanisms likely operate across the lifespan, with cumulative effects eventually resulting in AD phenotypes.

Extensive proteome remodeling has been documented during AD progression [40], and additional protein aggregates, beyond Aβ and Tau, may form in DS brains. Although TDP-43 pathology was rare and mostly observed in younger DS individuals [41], we and others have shown that SOD1 triplication [14] can result in pathological aggregation, similar to ALS[42]. SOD1 in DS is also oxidatively modified, increasing its aggregation propensity [43]. This supports the idea that loss of proteostasis, marked by protein misfolding, aggregation, and impaired clearance, is a defining molecular feature of early-onset AD in DS. Multiple mechanisms likely drive this proteostatic failure, including abnormal endosomal phenotypes [44, 45], impaired autophagy due to hyperactive mTOR signaling [15, 46], and dysregulated unfolded protein response (UPR) [47]. These abnormalities may accelerate or even initiate AD in DS through a complex interplay of genetic predisposition and downstream molecular disruptions [48].

Beyond proteostasis, DS cells also exhibit increased energy demand and mitochondrial dysfunction [49]. Mitochondrial defects in DS encompass altered dynamics, ultrastructural abnormalities, inefficient OXPHOS, elevated ROS production, and reduced ATP synthesis [16, 50–53]. Our data highlight widespread dysregulation of mitochondrial proteins, identifying this module as one of the most affected by trisomy 21. These findings align with studies in DS fibroblasts, iPSC models, and transgenic mice [54–57]. Similar mitochondrial abnormalities are also seen in other neurodevelopmental disorders with intellectual disability, such as Rett and Fragile X syndromes [58, 59], as well as in neurodegenerative diseases, including AD, Parkinson’s disease (PD), and Huntington’s disease (HD). In DS, mitochondrial dysfunction likely contributes both to early neurodevelopmental deficits and to age-related neurodegeneration.

Our parallel analysis of the Ts66Yah mouse model [21, 36], revealed substantial overlap in protein modules upregulated in human DS. Shared changes included disruptions in neurotransmission, insulin signaling, and mitochondrial function. However, unlike human DSAD samples, Ts66Yah mice did not show a global age-related reduction in DEPs at 9 months, likely reflecting the absence of a pronounced AD phenotype due to species-specific differences, including the non-amyloidogenic processing of murine APP.

Comparative analysis of DEPs in aged DS and control groups allowed us to examine age-related proteome remodeling relevant to AD. Both groups exhibited a decline in total DEPs with age, but this reduction was more pronounced in DSAD, indicating a heightened aging effect in trisomic individuals. These trends are consistent with recent single-nucleus transcriptomic studies in DSAD and sporadic AD brains, which revealed region-specific, transient disease processes [5]. Comparison of DEPs from our DSAD cohort with those from sporadic AD frontal cortex datasets [33] showed substantial overlap, supporting the view that DS represents a genetic form of AD. Pathway analysis in DSAD highlighted broadly negative *z*-scores across neurotransmission, stress responses, and developmental signaling.

Interestingly, aging effects in DS and controls showed convergence, with both groups displaying pathway inhibition in mitochondrial function, mTOR/UPR signaling, redox homeostasis, and synaptogenesis. These findings are consistent with studies showing that protein synthesis declines with age in various species and tissues [60, 61]. DS has also been proposed as a segmental progeroid syndrome [62, 63] with individuals experiencing multimorbidity and pathological aging*.* In aging groups, we observed reduced activity in NRF2-mediated oxidative stress responses, chaperone-mediated autophagy, and synaptogenesis pathways. However, unexpectedly, mitochondrial dysfunction and sirtuin signaling showed positive *z*-scores. This may reflect partial compensatory mechanisms, potentially reversing early-life trends. The reduced NRF2 activity with age may indicate a vanishing protective response, leading to the accumulation of oxidative damage [64].

## Conclusions and Limitations of the Study

Our proteomic analysis demonstrates that trisomy 21 significantly alters brain protein expression, with DS individuals exhibiting a general increase in DEPs compared to neurotypical controls. Pathway analysis revealed dysregulation across multiple systems, including metabolism, stress response, neurotransmission, synaptogenesis, and sirtuin signaling, defining a unique proteomic profile for DS. Importantly, individuals with DS and those with DSAD exhibit distinct proteomic signatures associated with AD pathology. Loss of proteostasis appears to play a central role, contributing to decreased protein expression, impaired mitochondrial function, and reduced NRF2-mediated stress responses. These changes likely exacerbate Aβ and tau pathology, promoting early accumulation of plaques and tangles in DS brains.

Despite these insights, some limitations must be acknowledged. First, the study was constrained by the small number of postmortem brain samples available, which may limit statistical power. Nevertheless, the observed protein expression patterns show substantial overlap with findings from previous studies, enhancing the robustness of our conclusions. Second, the analysis was restricted to the frontal cortex; other brain regions, such as the hippocampus, were not examined. Third, post-translational modifications and protein–protein interactions, which are critical for protein function, were not captured in this bottom-up proteomic approach. Additionally, low-abundance proteins, isoforms, and membrane proteins remain underrepresented due to methodological limitations. Finally, while our study captures proteomic changes before and after the onset of AD neuropathology in DS, Braak staging data were unavailable, precluding direct correlation with disease severity. Despite these limitations, this is the first study to characterize proteome-wide alterations in DS across aging and AD development, providing valuable insights into the molecular mechanisms driving neurodegeneration in this population.

## Supplementary Information

Below is the link to the electronic supplementary material.

ESM1 (DOCX 30.9 KB)

ESM2 (XLSX 878 KB)

ESM3 (XLSX 270 KB)

ESM4 (XLSX 197 KB)

ESM5 (XLSX 204 KB)

ESM6 (XLSX 17.2 KB)

ESM7 (XLSX 48.5 KB)

ESM8 (XLSX 348 KB)

ESM9 (XLSX 386 KB)

ESM10 (XLSX 138 KB)

ESM11 (XLSX 32.6 KB)

ESM12 (XLSX 22.4 KB)

ESM13 (XLSX 26.7 KB)

ESM14 (XLSX 27.3 KB)

ESM15 (XLSX 48.0 KB)

ESM16 (XLSX 309 KB)

ESM17 (XLSX 165 KB)

ESM18 (PPTX 45.7 MB)

## Data Availability

All processed data are available in the Supplementary Tables. Additional datasets generated in the current study are available from the corresponding author on reasonable request.

## Abbreviations

AD: Alzheimer’s disease
Aβ: Amyloid beta
AMPK: 5′ AMP-activated protein kinase
APP: Amyloid precursor protein
ATP5P B/O: ATP synthase peripheral stalk membrane subunit B / subunit OSCP
ATP6V1G2: ATPase H + transporting V1 subunit G2
CBR1: Carbonyl reductase 1
CTRY: Controls young
CTRO: Controls old
DEGs: Differentially expressed genes
DEPs: Differentially expressed proteins
DNM1L: Dynamin-1-like protein
DSAD: Down syndrome subject with Alzheimer’s disease
DS: Down syndrome
EEF: Eukaryotic elongation factor
FDR: False discovery rate
GAP43: Growth-associated protein 43
GFAP: Glial fibrillary acidic protein
HD: Huntington’s disease
HSA21: Human chromosome 21
IPA: Ingenuity pathway analysis
iPSC: Induced pluripotent stem cell
MAG: Myelin-associated glycoprotein
MAPT: Microtubule-associated protein tau
nLC-HDMSE: Nanoscale liquid chromatography high-definition expression configuration mode
NRF2: Nuclear factor erythroid 2-related factor 2
mTOR: Mammalian target of rapamycin
OPA1: Dynamin-like GTPase
OXPHOS: Oxidative phosphorylation
PD: Parkinson’s disease
PMI: Postmortem interval
ROS: Reactive oxygen species
S100B: S100 calcium-binding protein B
SLC1A3: Solute carrier family 1 member 3
SNAP91: Synaptosome-associated protein 91
SOD 1 and 2: Superoxide dismutase 1 (cytoplasmic) and 2 (mitochondrial)
STRING: Search Tool for the Retrieval of Interacting Genes/Proteins
SYNJ1: Synaptojanin 1
SYP: Synaptophysin
TCA: Tricarboxylic acid cycle
TXN: Thioredoxin
UNIPROT: The universal protein resource
UPR: Unfolded protein response
VDAC1-3: Voltage-dependent anion selective channel 1–3
YWHAX: 14-3-3ζ
