# Supplementary material for: Proteome Signature of Alzheimer-Like Phenotypes in Frontal Cortices From Young and Old Individuals With Down Syndrome
Source: Mol Neurobiol. 2025 Nov 21;63(1):126. doi: 10.1007/s12035-025-05432-0 (PMC12638384; doi:10.1007/s12035-025-05432-0)
Supplement: Supplementary file 1 — (DOCX 30.9 KB) [file 12035_2025_5432_MOESM1_ESM.docx]

**Supplementary Table 1 :** Patients demographic data, AA= African American, Ca= Caucasian, In = Indian

| **Subjects** | **Brain Region** | **PMI** | **Age** | **Sex** | **Race** | **Cause of death** |
| --- | --- | --- | --- | --- | --- | --- |
| Control Y 1 | Frontal Cortex | 5,8 | 39 | Female | Unknown | Unknown |
| Control Y 2 | Frontal Cortex | 12 | 22.8 | Male | African American | Arrhythmia due to hypertrophy cardiomyopathy |
| Control Y 3 | Frontal Cortex | 8 | 33.1 | Male | Caucasian | Cardiac Arrhythmia |
| Control Y 4 | Frontal Cortex | 10 | 24.4 | Male | Caucasian | Multiple injuries |
| Control Y 5 | Frontal Cortex | 10 | 10.8 | Female | Caucasian | Asthma |
| Control Y 6 | Frontal Cortex | 14 | 19.8 | Male | Caucasian | Multiple injuries |
| **CTR Young** |  | **9.96 ± 2.88** | **24.9 ± 9.95** | **4M, 2F** | **1AA, 4 Ca, 1 Un** |  |
| DS 1 | Frontal Cortex | 12 | 1.9 | Male | Caucasian | Unknown |
| DS 2 | Frontal Cortex | 14 | 15.5 | Male | Caucasian | Chromosome disorder, Trisomy 21 |
| DS 3 | Frontal Cortex | 10 | 39.6 | Male | African American | HCVD (Hypertensive Cardiovascular Disease) |
| DS 4 | Frontal Cortex | 12 | 39.2 | Female | Caucasian | Cancer |
| DS 5 | Frontal Cortex | 13 | 44.5 | Female | Caucasian | Cardiac Arrhythmia |
| DS 6 | Frontal Cortex | 14 | 19.9 | Male | Indian | Cardiopulmonary arrest: congenital heart disease |
| **DS** |  | **12.5 ± 1.51** | **26.7 ± 16.8** | **4M, 2F** | **1AA, 4 Ca, 1 In** |  |
| DS-AD 1 | Frontal Cortex | 5.3 | 57 | Female | Unknown | Seizure Disorder |
| DS-AD 2 | Frontal Cortex | 3 | 63 | Female | Unknown | Respiratory |
| DS-AD 3 | Frontal Cortex | 6 | 63 | Female | Unknown | Unknown |
| DS-AD 4 | Frontal Cortex | 4.5 | 55 | Male | Unknown | Pneumonia |
| DS-AD 5 | Frontal Cortex | 10.5 | 61 | Male | Unknown | Unknown |
| DS-AD 6 | Frontal Cortex | 3 | 57 | Female | Unknown | Pneumonia |
| **DS AD** |  | **5.4 ± 2.8** | **59.3 ± 3.44** | **2M, 4F** | **6 Un** |  |
| Control O 1 | Frontal Cortex | 5 | 47.3 | Female | Caucasian | Pneumonia |
| Control O 2 | Frontal Cortex | 8 | 64 | Female | Unknown | Myocardial infarction |
| Control O 3 | Frontal Cortex | 17 | 56.8 | Male | Caucasian | HACVD (Hypertensive Arteriosclerotic Cardiovascular Disease) |
| Control O 4 | Frontal Cortex | 16 | 55.3 | Male | Caucasian | Arteriosclerotic Cardiovascular Disease |
| Control O 5 | Frontal Cortex | 4.5 | 65 | Male | Unknown | Cardiac Arrest |
| Control O 6 | Frontal Cortex | 2.7 | 67 | Male | Unknown | Cardiomyopathy |
| **Control Old** |  | **8.9 ± 6.2** | **59.2 ± 7.48** | **4M, 2F** | **3Ca, 3Un** |  |
