# Supplementary material for: Proteome Signature of Alzheimer-Like Phenotypes in Frontal Cortices From Young and Old Individuals With Down Syndrome
Source: Mol Neurobiol. 2025 Nov 21;63(1):126. doi: 10.1007/s12035-025-05432-0 (PMC12638384; doi:10.1007/s12035-025-05432-0)
Supplement: Supplementary file 18 — (PPTX 45.7 MB) [file 12035_2025_5432_MOESM18_ESM.pptx]

## Slide 1
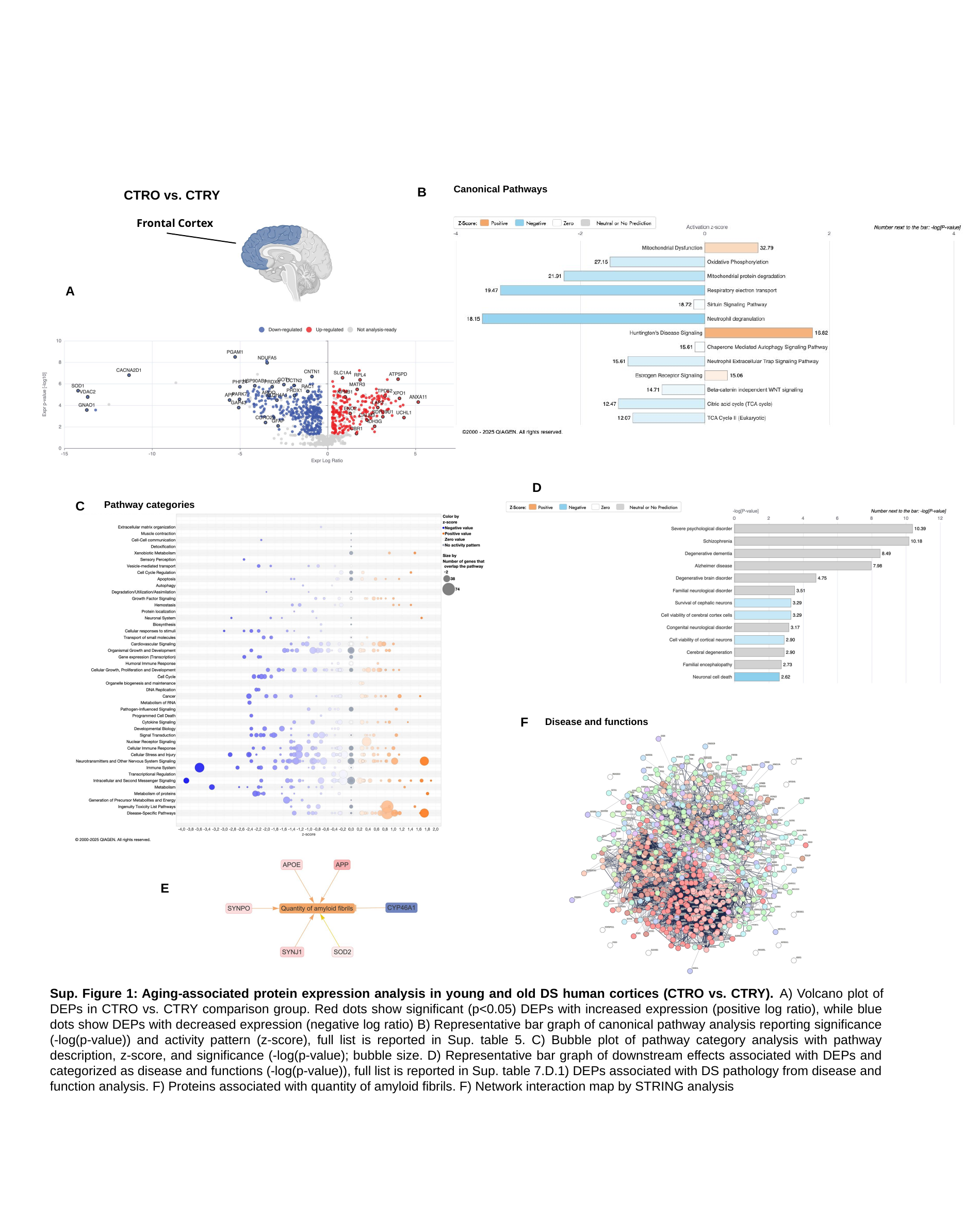

Canonical Pathways
B
CTRO vs. CTRY
Frontal Cortex
A
D
C
Pathway categories
F
Disease and functions
E
Sup. Figure 1: Aging-associated protein expression analysis in young and old DS human cortices (CTRO vs. CTRY). A) Volcano plot of DEPs in CTRO vs. CTRY comparison group. Red dots show significant (p<0.05) DEPs with increased expression (positive log ratio), while blue dots show DEPs with decreased expression (negative log ratio) B) Representative bar graph of canonical pathway analysis reporting significance (-log(p-value)) and activity pattern (z-score), full list is reported in Sup. table 5. C) Bubble plot of pathway category analysis with pathway description, z-score, and significance (-log(p-value); bubble size. D) Representative bar graph of downstream effects associated with DEPs and categorized as disease and functions (-log(p-value)), full list is reported in Sup. table 7.D.1) DEPs associated with DS pathology from disease and function analysis. F) Proteins associated with quantity of amyloid fibrils. F) Network interaction map by STRING analysis

## Slide 2
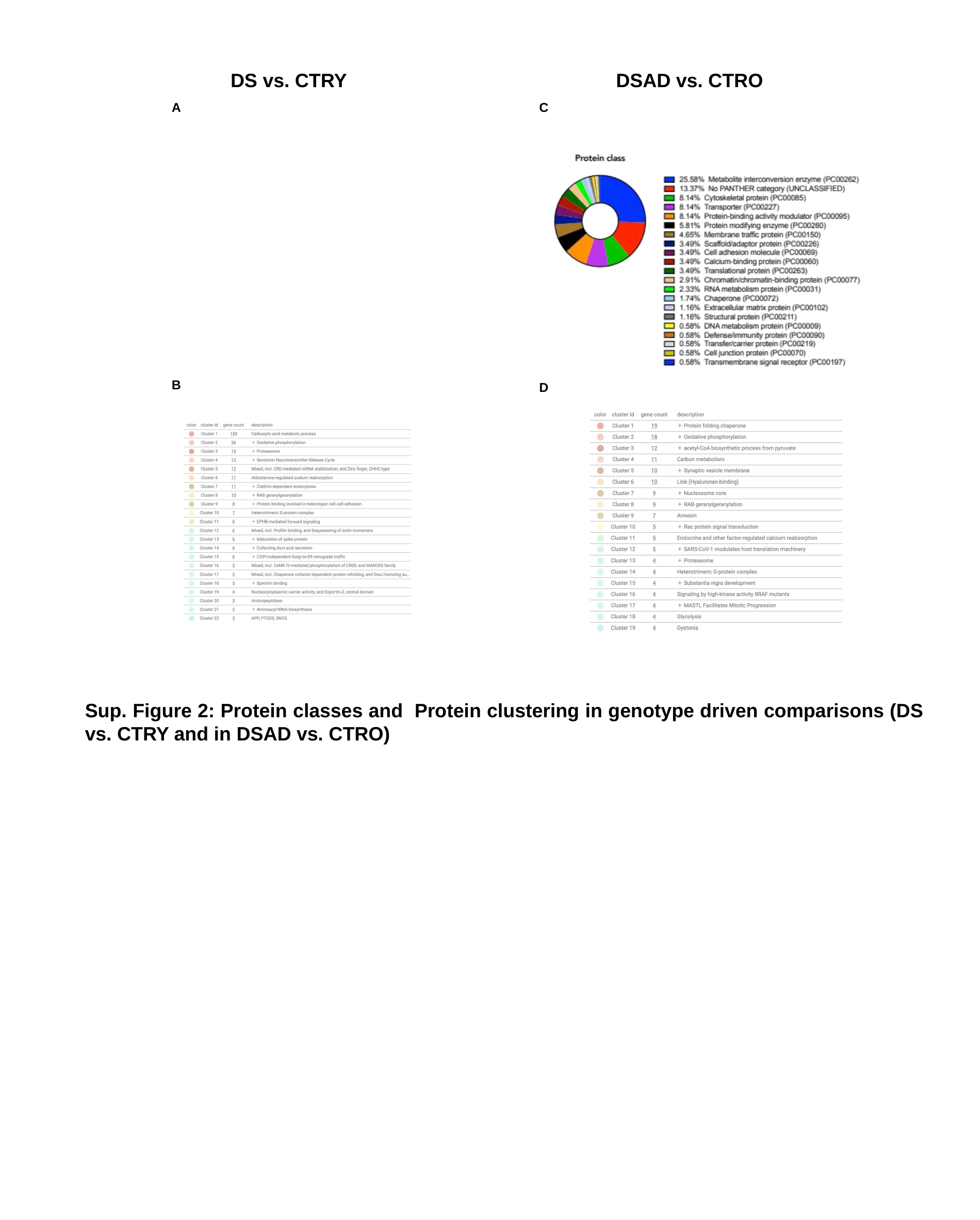

DS vs. CTRY
DSAD vs. CTRO
C
A
B
D
Sup. Figure 2: Protein classes and Protein clustering in genotype driven comparisons (DS vs. CTRY and in DSAD vs. CTRO)

## Slide 3
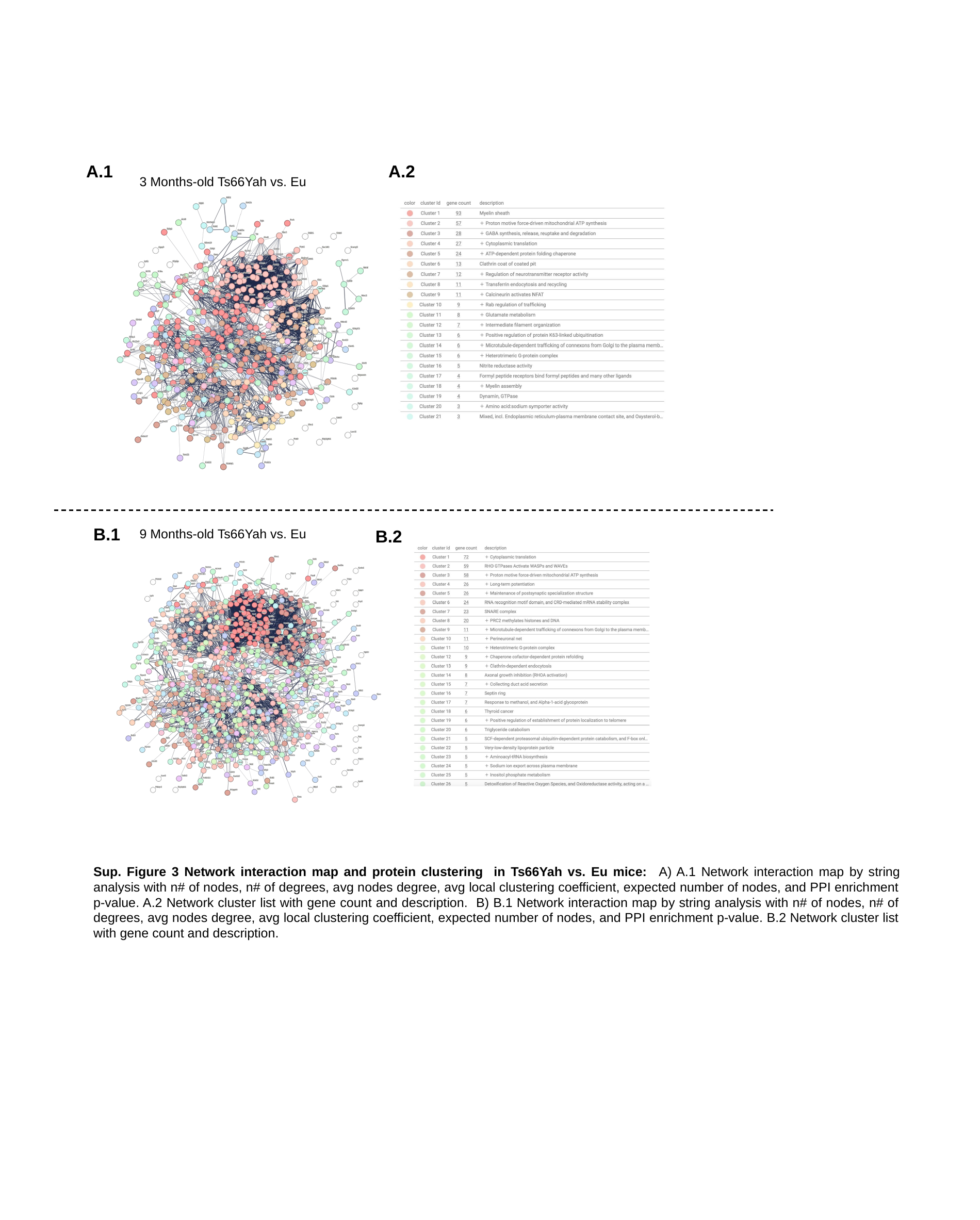

A.1
A.2
3 Months-old Ts66Yah vs. Eu
B.1
B.2
9 Months-old Ts66Yah vs. Eu
Sup. Figure 3 Network interaction map and protein clustering in Ts66Yah vs. Eu mice: A) A.1 Network interaction map by string analysis with n# of nodes, n# of degrees, avg nodes degree, avg local clustering coefficient, expected number of nodes, and PPI enrichment p-value. A.2 Network cluster list with gene count and description. B) B.1 Network interaction map by string analysis with n# of nodes, n# of degrees, avg nodes degree, avg local clustering coefficient, expected number of nodes, and PPI enrichment p-value. B.2 Network cluster list with gene count and description.

## Slide 4
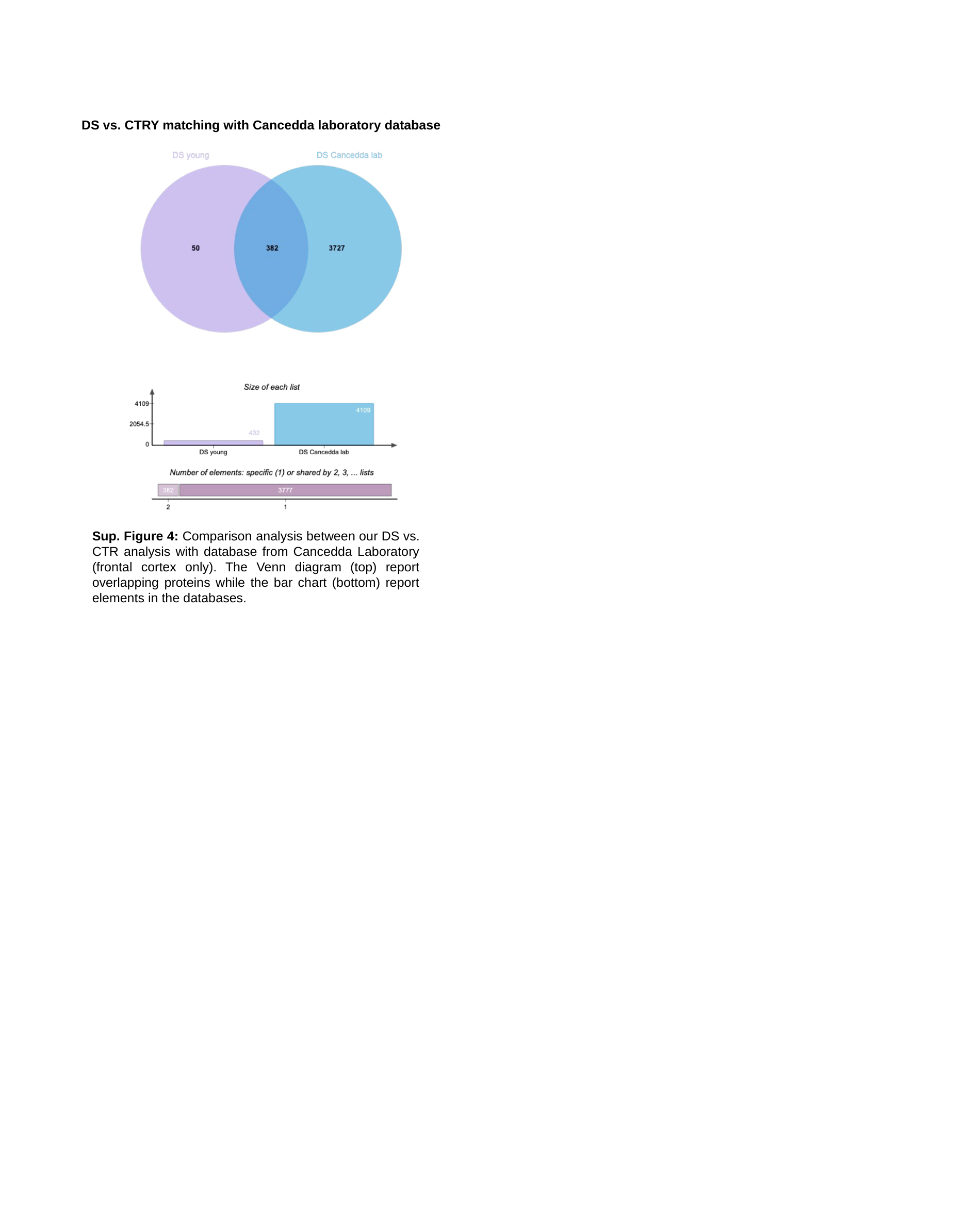

DS vs. CTRY matching with Cancedda laboratory database
Sup. Figure 4: Comparison analysis between our DS vs. CTR analysis with database from Cancedda Laboratory (frontal cortex only). The Venn diagram (top) report overlapping proteins while the bar chart (bottom) report elements in the databases.

## Slide 5
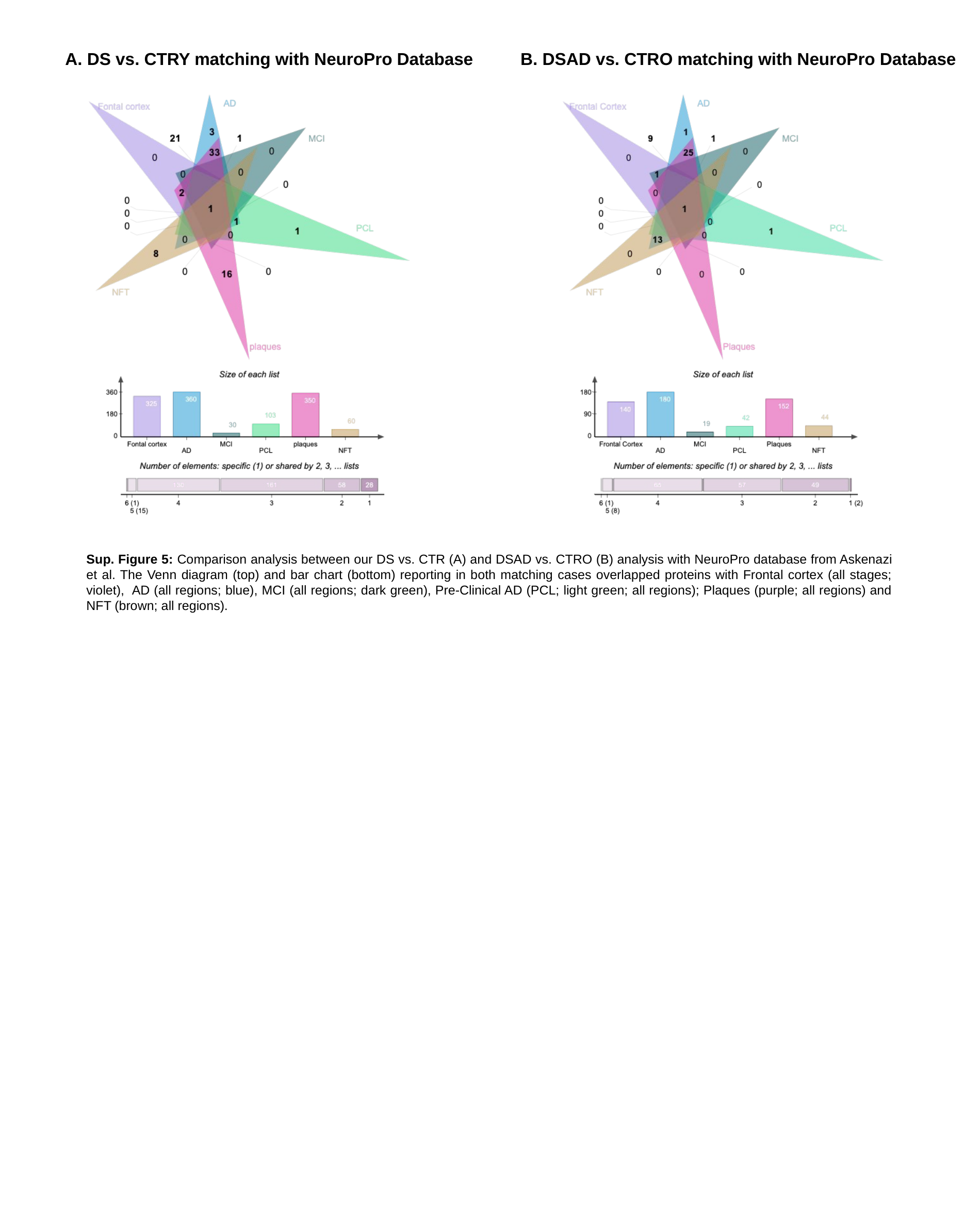

A. DS vs. CTRY matching with NeuroPro Database
B. DSAD vs. CTRO matching with NeuroPro Database
Sup. Figure 5: Comparison analysis between our DS vs. CTR (A) and DSAD vs. CTRO (B) analysis with NeuroPro database from Askenazi et al. The Venn diagram (top) and bar chart (bottom) reporting in both matching cases overlapped proteins with Frontal cortex (all stages; violet), AD (all regions; blue), MCI (all regions; dark green), Pre-Clinical AD (PCL; light green; all regions); Plaques (purple; all regions) and NFT (brown; all regions).
